# Age at Menarche, Level of Education, Parity and the Risk of Hysterectomy: A Systematic Review and Meta-Analyses of Population-Based Observational Studies

**DOI:** 10.1371/journal.pone.0151398

**Published:** 2016-03-10

**Authors:** Louise F. Wilson, Gita D. Mishra

**Affiliations:** The University of Queensland, Centre for Longitudinal and Life Course Research, School of Public Health, Public Health Building, Herston Road, Herston, Queensland, 4006, Australia; NHS lothian and University of Edinburgh, UNITED KINGDOM

## Abstract

**Background:**

Although rates have declined, hysterectomy is still a frequent gynaecological procedure. To date, there has been no systematic quantification of the relationships between early/mid-life exposures and hysterectomy. We performed a systematic review and meta-analyses to quantify the associations between age at menarche, education level, parity and hysterectomy.

**Methods:**

Eligible studies were identified by searches in PubMed and Embase through March 2015. Study-specific estimates were summarised using random effects meta-analysis. Heterogeneity was explored using sub-group analysis and meta-regression.

**Results:**

Thirty-two study populations were identified for inclusion in at least one meta-analysis. Each year older at menarche was associated with lower risk of hysterectomy—summary hazard ratio 0.86 (95% confidence interval: 0.78, 0.95; *I*^*2*^ = 0%); summary odds ratio 0.88 (95% confidence interval: 0.82, 0.94; *I*^*2*^ = 61%). Low education levels conferred a higher risk of hysterectomy in the lowest versus highest level meta-analysis (summary hazard ratio 1.87 (95% confidence interval: 1.25, 2.80; *I*^*2*^ = 86%), summary odds ratio 1.51 (95% confidence interval: 1.35, 1.69; *I*^*2*^ = 90%)) and dose-response meta-analysis (summary odds ratio 1.17 (95% confidence interval: 1.12, 1.23; *I*^*2*^ = 85%) per each level lower of education). Sub-group analysis showed that the birth cohort category of study participants, the reference category used for level of education, the year the included article was published, quality of the study (as assessed by the authors) and control for the key variables accounted for the high heterogeneity between studies in the education level meta-analyses. In the meta-analyses of studies of parity and hysterectomy the results were not statistically significant.

**Conclusions:**

The present meta-analyses suggest that the early life factors of age at menarche and lower education level are associated with hysterectomy, although this evidence should be interpreted with some caution due to variance across the included studies.

## Introduction

Hysterectomy continues to be one of the most frequent gynaecological procedures performed in more economically developed countries [1, 2] and has been associated with lower quality of life [3, 4] and poorer health outcomes [5–7]. Approximately 20–45% of women in these countries will have a hysterectomy by the time they are between 60–70 years of age [8–10], with the majority performed for benign indications such as uterine fibroids, dysfunctional uterine bleeding, prolapse and endometriosis [2].

With such high prevalence, it is important to understand the exposures that occur in earlier life that might predict the risk of hysterectomy to alert practitioners of the need to proactively monitor these women for the benign indications of hysterectomy and implement appropriate treatment pathways. Lower socio-economic status (SES), particularly lower levels of education, have been associated with a higher risk of hysterectomy, however, this varies by geographic location [11, 12], ethnicity [13–15] and birth cohort [16]. The associations between a range of reproductive factors and hysterectomy have also been studied. In general, an earlier age at menarche [17–19] and age at first birth [18, 20] have been associated with an increased risk of hysterectomy; while the associations with other adult reproductive factors such as parity [18, 21] and number of miscarriages [19, 22] are less clear.

To date however, there has been no systematic quantification of the relationships between these early/mid-life exposures and hysterectomy. The aim of our study therefore was to systematically review and quantify the evidence from observational studies, through meta-analysis, on the relationship between SES and reproductive factors and hysterectomy.

## Materials and Methods

The systematic review and meta-analyses were done in accordance with the Preferred Reporting Items for Systematic Reviews and Meta-analyses (PRISMA) Guidelines [23, 24]. An official protocol, however, was not published or registered, although the strategy for this review was discussed and agreed. The clinical question posed was: what is the relationship between SES and reproductive factors and hysterectomy. Meta-analysis was possible for three exposures: age at menarche, level of education and parity.

### Search Strategy

We conducted three separate systematic searches in Embase and PubMed: 1) age at menarche, 2) SES, and 3) adult reproductive factors and hysterectomy. The databases were searched for records from inception until March 2015. Full details of the three search strategies are available online in S1 File: Search Strategy. For all searches we used both Medical Subject Headings (MeSH terms) and text words in PubMed, and Emtree terms and text words in Embase. No language or publication data restrictions were imposed. Reference lists of articles were checked for additional articles.

### Study selection

One investigator (L.W.) did the initial screen of titles and the abstracts of articles identified through the initial screening process. Two investigators (L.W. and G.M.) independently reviewed the full text of potentially relevant articles for final inclusion, with any disagreement resolved through discussions.

Articles were eligible for inclusion if they 1) reported measures of association and 95% confidence intervals (CI) (or the raw data to calculate these) for associations between any SES factor, age at menarche or adult reproductive factor and hysterectomy, and 2) had population-based samples or cohorts. Studies that focused on population sub-groups (e.g. veterans), or used hospital-based controls, were excluded.

We also contacted several authors [17, 20, 25] for additional information or results. All responded; with two [20, 25] able to provide some or all of the requested information.

### Quality evaluation

Study quality was assessed independently by L.W. and G.M. using the Newcastle-Ottawa Scale for cohort studies. The scale includes 9 items under three categories: selection, comparability and outcome. Three of the items were not relevant to assessment of cross-sectional studies (outcome not present at start of study, sufficient follow-up for outcome to occur, and adequacy of cohort follow-up). Unique scales were developed for each review as elements of the selection and comparability sections are exposure specific (available online in S2 File: Quality Assessment Scales).

### Data extraction

Data was extracted by L.W. and independently checked by G.M. for accuracy and completeness. Any disagreements were resolved through discussion. For each study we extracted: first author, year of publication, country of study, study design, study description, survey/baseline year, length of follow-up (cohort studies only), age group of sample (from which we derived the birth cohort of the sample), prevalence of hysterectomy in sample, outcome/comparator groups, measures of association (for the relevant exposure variables) and the covariates used in the model.

### Statistical analysis

For all meta-analyses, measures of association were summarised using a random-effects model due to the variability in characteristics between studies. The majority of studies reported either hazard ratios (HR) or odds ratios (OR). We summarised these two types of measures in separate meta-analyses. When articles reported measures of association with different covariates, the most fully-adjusted model was included in the meta-analysis.

#### Lowest versus Highest Education Level

We conducted meta-analyses on all eligible studies, and also those that only reported adjusted results. If the reference category in the published study was the lowest education level, the inverse of the HR/OR, and the upper and lower confidence intervals, were calculated for inclusion.

#### Dose-response meta-analyses

Studies were eligible for inclusion if they reported a dose-response association or categorical variable with at least three exposure levels. Dose-response measures of association were calculated for each study assuming a log-linear relationship between the exposure and hysterectomy. For the education meta-analysis, the lowest education level category had to be equivalent to “high school or less” and the highest education level category equivalent to “university/college degree or higher”. Highest education level was used as the reference category. Where the lowest education level was used as the reference category, the inverse of the calculated dose-response effect estimates and 95% confidence intervals were used. The HR/OR for a one category change in education level was assumed to approximate the HR/OR for one level lower in education.

For age at menarche, the HR/OR for a one category increase was assumed to approximate a per year increase in age at menarche; while for parity, this approximated the HR/OR for a per child increase (with no children as the reference category).

#### Exploration of heterogeneity

Between-study heterogeneity was assessed using the chi^2^ (Cochrane Q) and *I*^*2*^ statistics. We considered *I*^*2*^ values of 25%, 50% and 75% as cut-off points for evidence of low, moderate and high heterogeneity respectively [26]. Meta-regression and sub-group analyses were undertaken for the level of education meta-analyses if at least ten studies were included [27]. For the age at menarche and parity meta-analyses, where there were too few studies for meta-regression, sensitivity analyses were conducted omitting each study in turn to assess whether any single study contributed significantly to heterogeneity.

A priori study characteristics considered in our analysis of heterogeneity in the education meta-analyses included: year of publication (before 2000; 2000 and later), study design (prospective or cross-sectional), whether the definition of hysterectomy included, excluded or did not specify women with malignant conditions, the region in which the study population was selected (United States, Europe/United Kingdom, Australia/New Zealand, Asia), birth cohort of the study population (pre-1945, baby-boomers (1945–1965), broad population cohort), and cut-off for highest education level (high school, college/university degree, other). Post hoc, on the basis of additional expert input, prevalence of hysterectomy, adjustment for key confounding variables (identified through the quality assessment—see S2 File: Quality Assessment Scales) and quality of study (see online S5 File: Quality Assessment) were also considered.

We first undertook univariable random-effects meta-regression to investigate the role of each of the study characteristics described above on between-study heterogeneity. Where variables had more than one category overall effects were assessed (with 1000 permutations). Characteristics with a p-value of ≤0.2 were included in the multivariable model [28, 29].

Publication bias was assessed when there were at least ten studies included in the meta-analysis[27]. This was done through visual inspection of a funnel plot and through formal assessment undertaken by performing both Egger’s test and Begg and Mazumdar’s Rank Correlation test.

All analyses were conducted in Stata version 12.1.

## Results

### Literature search

**Fig 1:**
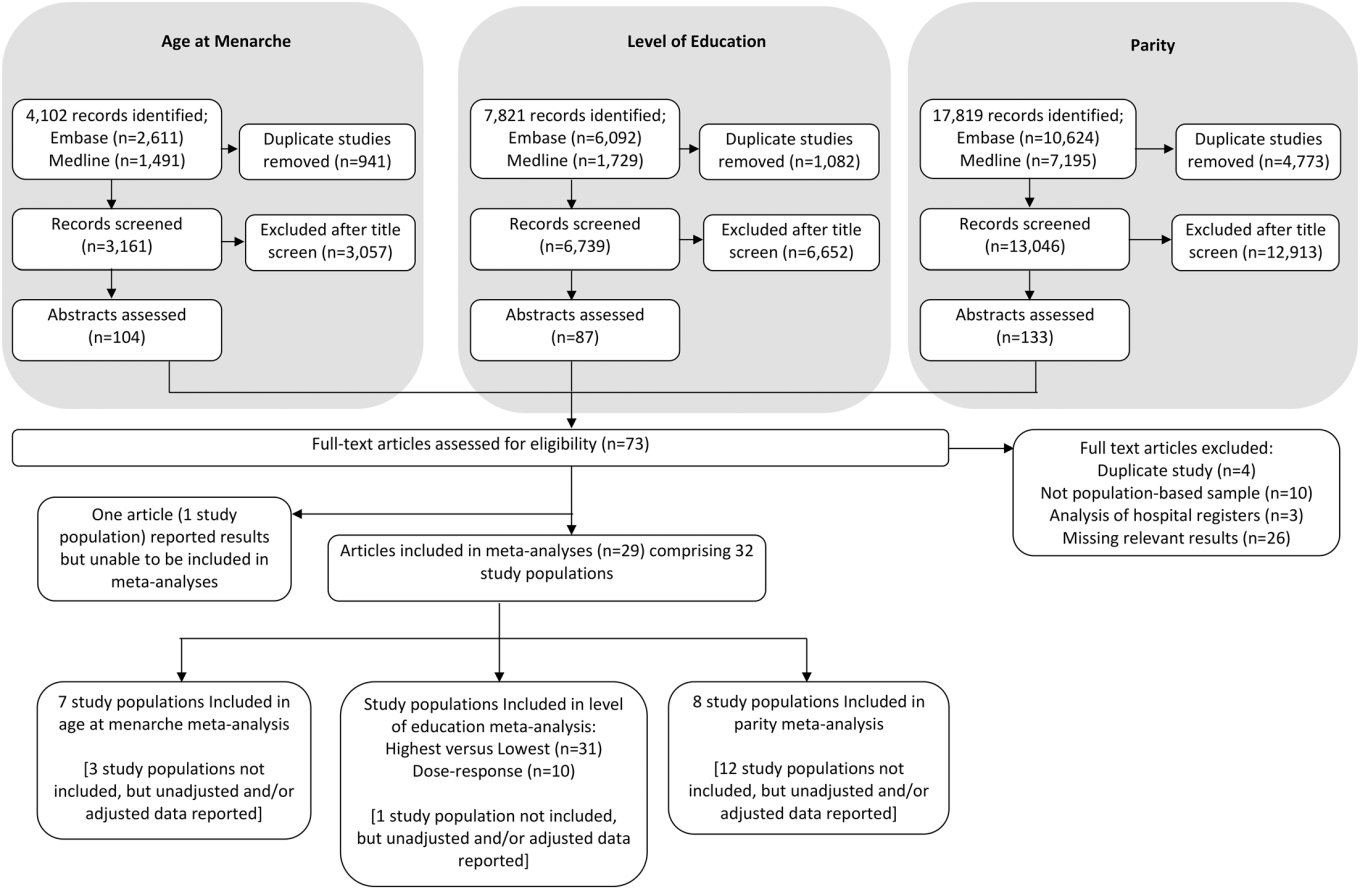
Flow chart detailing the search for and selection of studies in the age at menarche, level of education and parity meta-analyses. Studies were published up until March 2015.

The numbers of identified and included articles and study populations are summarised in Fig 1. Meta-analysis was possible for three exposures: age at menarche, level of education and parity. For other SES variables (occupation, employment and income) and adult reproductive factors (age at first birth, number of pregnancies and number of miscarriages) there were either an insufficient number of studies and/or incomparable categories or populations for a meta-analysis to be performed. Across the three searches, 4,102, 7,821 and 17,819 records were identified for age at menarche, SES and reproductive factors respectively. After excluding duplicates and undertaking an initial screen of titles, 104 article abstracts were reviewed for age at menarche, 87 article abstracts for SES factors and 133 for reproductive factors. Across the three searches there was overlap in the full-text articles identified for complete assessment. No non-English language papers were eligible for inclusion. In total, the full-texts of 73 articles were obtained. Of these, 29 articles [11–22, 25, 30–45] with results from 32 study populations contributed data to one or more meta-analysis. The age at menarche meta-analysis included seven study populations [17–19, 30–32]; the lowest versus highest education meta-analysis included 31 study populations [11–16, 20–22, 25, 30–45]; the dose-response education level meta-analysis included ten study populations [14, 16, 32, 39–42, 45], and the parity meta-analysis included eight study populations [11, 12, 14, 18, 19, 21, 44, 45].

### Study characteristics

The characteristics of the 32 study populations that were included in at least one of the meta-analyses are summarised in Table 1.

**Table 1:** Description of studies meeting inclusion criteria for age at menarche, level of education and parity meta-analyses. Abbreviations: LH = lowest versus highest; DR = dose-response.

First Author (year)[reference]	Meta-analysis	Design	Country of Study and Study Population	Baseline/ Survey year (years of follow-up)	Age range (birth cohort)	Sample (% hysterectomy)	Outcome/ comparator	Co-variates included in model
Bower(2009)[17]	Age at menarche	Cross-sectional	**United States**. Coronary Artery Risk Development in Young Adults (**CARDIA**) study. Black and White participants selected from four diverse sites across the US (Birmingham, Chicago, Minneapolis and Oakland)	2000–2002	33–45 (1955–1967)	1,863(10%)	Women with a hysterectomy with or without oophorectomy/Women without a hysterectomy	**Age at menarche**: race, age, years of education, access to medical care, BMI, polycystic ovarian disease/syndrome, tubal ligation, depressive symptoms, geographic site
Brett (1997)[33]	Education (L H)	cohort	**United States**. National Health and Nutrition Examination Survey (**NHNES**).Representative sample of the civilian US population.	1971–1975 (17–21 years)	<50 (1922–1950)	3,526 (22%)	Women with a hysterectomy/Women without a hysterectomy	**Education (LH)**: age, race, age at first birth, no. of miscarriages
Brett (2003)[39]	Education (L H)Education (DR)	cross-sectional	**United States**. National Health Interview Surveys 1998/1999 (**NHHS1998**). Multipurpose health survey of the civilian non-institutionalised US population, with over-sampling of Hispanic and Black households. Hysterectomy prevalence and odds ratios included in meta-analysis are for white women only.	1998–1999	>25 (pre 1974)	20,607 (23%)	Non-Hispanic white women with a hysterectomy/Non-Hispanic white women without a hysterectomy	**Education (LH)/Education (DR)**: age at interview, marital status, family income relative to US poverty line, region of current residence, BMI, whether had usual source of Medicare, self-reported health status, interaction between age and race
Ceausu (2006)[40]	Education (L H)Education (DR)	cross-sectional	**Sweden**. Women's Health in the Lund Area (**WHLA**) Study. All women born between December 2, 1935 and December 1, 1945 and living in the Lund area by December 1, 1995, identified through a population register of all inhabitants.	1995	50–60 (1935–1945)	6,917 (12%)	Women who reported hysterectomy / women without hysterectomy	**Education (LH)/Education (DR)**: working status
Cooper (2005)[34]	Education (L H)	cross-sectional	**United Kingdom**. Aberdeen Children of the 1950s Study (**Aberdeen**). Cohort born in Aberdeen between January 1950 and December 1955 who attended Aberdeen primary schools and participated in surveys in 1962 and were successfully traced in 1999.	2000/01	45–51 (1950–1955)	3,208 (14%)	Women with a hysterectomy (with or without oophorectomy)/ Women without a hysterectomy (with or without oophorectomy)	**Education (LH)**: own social class, housing, access to care
Cooper(2005)[34]	Education (L H)	cross-sectional	**United Kingdom**. British Women's Heart and Health Study (**BWHHS**). Participants selected randomly from general practitioner lists in 23 British towns.	1999–2001	60–79 (1919–1940)	3,208 (22%)	Women with a hysterectomy and/or oophorectomy / Women without a hysterectomy (with or without oophorectomy)	**Education (LH)**: own social class, housing, access to care
Cooper (2008)[16]	Education (L H) Education (DR)	cross-sectional	**Australia**. Australian Longitudinal Study of Women's Health (**ALSWH-mid**) (mid-cohort). Random stratified sampling from Health Insurance Commission database, with rural and remote women selected at twice the rate of women in urban areas.	1996	45–50 (1946–1951)	14,078 (30%)	Women with a hysterectomy and/or oophorectomy / Women without a hysterectomy (with or without oophorectomy)	**Education LH**: age-adjusted. **Education DR**: unadjusted
Cooper (2008)[16]	Education (L H) Education (DR)	cross-sectional	**Australia**. Australian Longitudinal Study of Women's Health (**ALSWH-older**) (Older-cohort). Random stratified sampling from Health Insurance Commission database, with rural and remote women selected at twice the rate of women in urban areas.	1996	70–75 (1921–1926)	12,792 (37%)	Women with a hysterectomy and/or oophorectomy / Women without a hysterectomy (with or without oophorectomy)	**Education LH**: age-adjusted. **Education DR**: unadjusted
Cooper (2008)[16] Cooper (2008)[18]	Education (L H) Education (DR) Age at menarche Parity	cohort	**United Kingdom**. National Survey of Health and Development (**NSHD**). A socially stratified sample of singleton babies born to married parents during one week of March, 1946.	1946 (57 years)	57 (1946)	Education 1,518 (24%); Age at menarche/ Parity 1797 (22%)	Women with a hysterectomy and/or oophorectomy / Women without a hysterectomy	**Age at menarche**: parity, father’s occupational class, educational level attained, BMI. **Education (LH)**: age-adjusted. **Education (DR)**: unadjusted. **Parity**: age at menarche, father’s occupational class, educational level attained, BMI
Dennerstein (1994)[30]	Age at menarche Education (L H)	cross-sectional	**Australia**. Melbourne Women's Midlife Health Project (**MWMHP**). Participants selected randomly from a computerised database of Melbourne telephone numbers.	1991	45–55 (1936–1946)	1,890 (22%)	Women who had reported hysterectomy (without unilateral or bilateral oophorectomy)/ women without hysterectomy	**Age at menarche/Education (LH)**: age, premenstrual symptoms, number of DC, number of non-gynaecological operations, current use of HRT, use of 1 or more prescription medications, smoking
Dharmalingam (2000)[35]	Education (L H)	cross-sectional	**New Zealand**. Family Formation Survey (**FFS**). Participants selected with a stratified cluster sampling procedure, including over-sampling of Maori population.	1995	25–59 (1936–1970)	2,367 (11%)	Women with a hysterectomy / women without a hysterectomy [oophorectomy not mentioned]	**Education (LH)**: Calendar period of hysterectomy, age, ethnicity, parity, pregnancy loss, tubal sterilization, use of pill, use of IUD, IUD side effect, occupation, marital status
Erekson (2009)[41]	Education (L H) Education (DR)	cross-sectional	**United States**. Behavioral Risk Factor Surveillance Survey 2004 (**BRFSS2004**). Representative sample of households with telephones in 49 US states, the District of Columbia, Guam, Puerto Rico and the Virgin Islands.	2004	≥18 (pre-1986)	180,982 (26%)	Women with a hysterectomy / women without a hysterectomy (oophorectomy status unknown)	**Education (LH)/Education (DR)**: age at questionnaire, region of country, race, education level, annual household income, employment status
Harlow (1999)[21]	Education (L H) Parity	cross-sectional	**United States**. Women aged 36–44 years from seven Boston metropolitan area communities were identified from Massachusetts Town Books (**Mass.**).	1995–1997	36–44 (1951–1959)	4,278 (3%)	women who were surgically menopausal / women who were premenopausal	**Education (LH)/Parity**: age and other factors (these were not specified, however other covariates reported were race, marital status, BMI, smoking, age at menarche, history of irregular cycles, oral contraceptive use, history of pain with periods, history of endometriosis, history of uterine fibroids, removal of one ovary, combination of endometriosis, uterine fibroids or ovary removal)
Hautaniemi (2003)[13]	Education (L H)	cross-sectional	**United States**. Hispanic Health and Nutrition Examination Survey (**HHNES**). Multistage stratified cluster survey of Mexican-Americans living in the US Southwest, Cuban Americans in Dade County, Florida and Puerto-Rican residents of New York City. Study focused on Mexican-Americans.	1982–1984	20–74 (1908–1962)	1,868 (14%)	women with a history of hysterectomy (including with oophorectomy)/ women without a hysterectomy	**Education (LH)**: age, language preference, parity, education, poverty
Kjerulff (1993)[42]	Education (LH) Education (DR)	cross-sectional	**United States**. Behavioral Risk Factor Surveillance Survey 1988 (**BRFSS1988**).Participants selected through random digit dialling across 16 US states.	1988	25–54 (1934–1963)	7,139 (18%)	Women who had ever had a hysterectomy / women who had not had a hysterectomy	**Education (LH)/Education (DR)**: age
Koepsell (1980)[12]	Education (L H) Parity	cross-sectional	**United States**. Participants selected through area-stratified random-sampling methods of two Washington State counties (**Wash. State**).	1976	35–74 (1922–1941)	1,087 (33%)	women reporting prior hysterectomy/women without prior hysterectomy	**Education (LH)/Parity**: odds ratios calculated from raw counts
MacLennan (1993)[43]	Education (L H)	cross-sectional	**Australia**. South Australia Health Omnibus Survey (**SAHOS**). Participants selected through self-weighting, multistage, systematic, representative area cluster sampling of households in metropolitan and country South Australia.	1991	≥40 (pre-1951)	1,042 (28%)	Women who reported hysterectomy/ women without hysterectomy	**Education (LH)**: unadjusted
Marks (1997)[20]	Education (L H)	cohort	**United States**. Wisconsin Longitudinal Study (**WLS**). Random sample of Wisconsin high school graduates in 1957.	1957 (36 years)	53–54 (1939–1940)	3,326 (31%)	Women who had undergone a hysterectomy by age 54/women without a hysterectomy to age 54	**Education (LH)**: Only able to include unadjusted results in model (confidence intervals unable to be calculated for adjusted results)
Meilahn (1989)[15]	Education (L H)	cross-sectional	**United States**. Participants selected randomly from driver’s license lists of people resident in Pittsburgh, Pennsylvania (**Pitts.)–**Black women.	1983	40–52 (1931–1943)	326 (47%)	Women with a hysterectomy with or without oophorectomy, or oophorectomy alone/ women without hysterectomy or oophorectomy	**Education (LH)**: age, race, age at menarche, number of children, BMI, cigarette smoking, religion
Meilahn (1989)[15]	Education (L H)	cross-sectional	**United States**. Participants selected randomly from driver’s license lists of people resident in Pittsburgh, Pennsylvania (**Pitts.**)**–**White women.	1983	40–52 (1931–1943)	1,785 (24%)	Women with a hysterectomy with or without oophorectomy, or oophorectomy alone/ women without hysterectomy or oophorectomy	**Education (LH)**: age, race, age at menarche, number of children, BMI, cigarette smoking, religion
Nagata (2001)[11]	Education (L H) Parity	cohort	**Japan**. Takayama Health Study (**THS**). Random selection of participants still resident in Takayama City, Japan in 1998 and who had reported being pre-menopausal at baseline survey in 1992.	1992 (6 years)	35–54 (1938–1957)	1,172 (3%)	Women who reported premenopausal hysterectomy/women without hysterectomy	**Education (LH)/Education (DR)/Parity**: age, body size, smoking status, exercise habits, age at menarche, age at which regular menses started, age at birth of first child, history of abortion, intake of alcohol and macro- and micro-nutrient
Palmer (1999)[31]	Age at menarche Education (L H)	cross-sectional	**United States**. Black Women's Health Study (**BWHS**). Subscribers to *Essence* Magazine (a women’s magazine marketed to Black women), members of selected professional organisations and friends and relatives of respondents.	1995	30–49 (1946–1965)	34,950 (15%)	Women with a hysterectomy (including oophorectomy) /pre-menopausal women without a hysterectomy. Women with cancer of the cervix or uterus were excluded.	**Age at menarche/Education (LH)**: current age, geographic region, uterine leiomyoma, endometriosis, age at first birth, parity, tubal ligation
PMISG (2000)[44]	Education (L H) Parity	cross-sectional	**Italy**. Women attending a network of first-level outpatient menopause clinics in for general counselling about menopause or treatment of menopausal symptoms (**PSMIG**).	1997–1999	40–76 (1919–1955)	25,644 (18%)	Women with hysterectomy (with or without oophorectomy) for benign conditions /women without hysterectomy	**Education (LH)/Parity**: age, BMI
Powell (2005)[14]	Education (L H) Education (DR) Parity	cross-sectional	**United States**. Study of Women's Health Across the Nation (**SWAN**). Multi-centre, multi-ethnic study in 7 metropolitan US cities using a community-based sampling approach in a defined geographic area with either a list-based sampling frame, random-digit dialling or a combination of the two.	1995–1997	40–55 (1940–1955)	15,160 (19%)	Women with a hysterectomy for benign conditions/ women without a hysterectomy (excl. women with cancer of the uterus, cervix or ovary)	**Education (LH)/Education (DR)/Parity**: odds ratios calculated from raw counts of total sample (confidence intervals unable to be calculated from adjusted results)
Qi (2013)[45]	Education (L H) Education (DR) Parity	cross-sectional	**United States**. Women's Health Initiative (**WHI**). Self-identified African-American participants of the WHI (recruited from 40 clinical centres across the US).	1993–1998	50–79 (1914–1943)	10,439 (56%)	Women with self-report of hysterectomy at baseline/ women without hysterectomy	**Education (LH)/Education (DR)**: age at entry, African admixture, BMI, parity, age at menarche, years smoking, alcohol intake **Parity**: calculated from raw counts
Santow (1992)[22]	Education (L H)	cross-sectional	**Australia**. Australian Family Project (**AFP**). One-in-one thousand nationally representative probability sample of private dwellings in Australia identified approximately 5,000 households which were then screened to identify eligible women who were usual residents of the dwellings.	1986	20–59 (1927–1966)	2,547 (10%)	Women with prior history of hysterectomy/ women with intact uteri	**Education (LH)**: age group, parity, side effects IUD, use of pill, tubal sterilization, race, state, time period
Santow (1995)[36]	Education (L H)	cross-sectional	**Australia**. 3rd Risk Factor Prevalence Survey (**RFPS**) (Canberra component). Participants who agreed to take part in in-home interviews as follow-up to a survey and clinic check. Potential respondents of the initial survey and clinic check were identified from Commonwealth Electoral Rolls.	1992	20–59 (1933–1972)	276 (16%)	Women with a hysterectomy/ women without a hysterectomy	**Education (LH)**: age group, parity, side effects IUD, tubal sterilization, caesareans, menstrual problems
Schofield (1991)[37]	Education (L H)	cross-sectional	**Australia**. Participants in a community survey on pap-smear attitudes, selected through a Census District sampling framework in the Hunter Valley region of New South Wales (**HunterVall.**).	1987–1988	35–54 (1933–1952)	1,885 (10%)	Women who had a hysterectomy / women without a hysterectomy	**Education (LH)**: age-stratified
Settnes (1996)[38] Settnes (1997)[19]	Education (L H) Age at menarche Parity	Cohort	**Denmark**. Participants selected (using a random number generator) from the population in the Western part of Copenhagen County (**Copen_coh**).	1982–1984 (6–8 years)	30 and 40 (1942, 1952)	914 (4%)	women with a hysterectomy for benign conditions/ women without a hysterectomy (women with hysterectomy with malignant diagnosis were excluded)	**Age at menarche**: age-adjusted. **Education (LH)**: age. **Parity**: age, vocational education, abortions, oral contraceptives, progestogen-only minipill
Settnes (1996)[38] Settnes (1997)[19]	Education (LH) Age at menarche	cross-sectional	**Denmark**. Participants selected (using a random number generator) from the population in the Western part of Copenhagen County (**Copen_XS**).	1982–1984	30, 40, 50 and 60 (1922, 1932, 1942 and 1952)	1,737 (9%)	women with a hysterectomy for benign conditions/ women without a hysterectomy (women with hysterectomy with malignant diagnosis were excluded)	**Age at menarche**: age, schooling, vocational education, ascendant social status by marriage, parity, oral contraceptives. **Education (LH)**: age, vocational education, ascendant social status by marriage
Sievert (2013)[32]	Age at menarche Education (L H) Education (DR)	cross-sectional	**United States**. Hilo Women's Health Study (**HWHS**). Participants selected through postal surveys mailed to property lots in Hilo, Hawaii chosen by random assignment of tax map key numbers.	2005	40–60 (1945–1965)	898 (18%)	Women with a history of hysterectomy / women without a hysterectomy	**Age at menarche/Education (LH)/Education (DR)**: age, ethnicity, BMI at age 30, married 20 years ago, parity, current smoking
Stang (2014)[25]	Education (L H)	Cross-sectional	**Germany**. Pooled analysis of six German population-based cohorts drawn from random samples of mandatory residence lists (**German cohorts**).	1997–2006	20–84 (1916–1978)	9,536(19%)	Women with a history of hysterectomy/ women without a hysterectomy	**Education (LH)**: age, region

Of the identified study populations, five were cohort and 27 were cross-sectional. Study populations were drawn from nine countries—all were more economically developed locations. The United States was the most common study population setting (n = 14). Study population samples ranged from 276 to 180,982 participants, and hysterectomy prevalence from 3% to 56% (average 20%).

For each exposure, data on associations were reported for up to 12 study populations but were not included in our meta-analyses because they used a different reference category, exposure variables were categorized in ways that prevented comparison, or there were an insufficient number of categories to enable a dose-response association to be calculated (details of these study populations are available online in S3 File: Tables).

### Age at menarche

**Fig 2:**
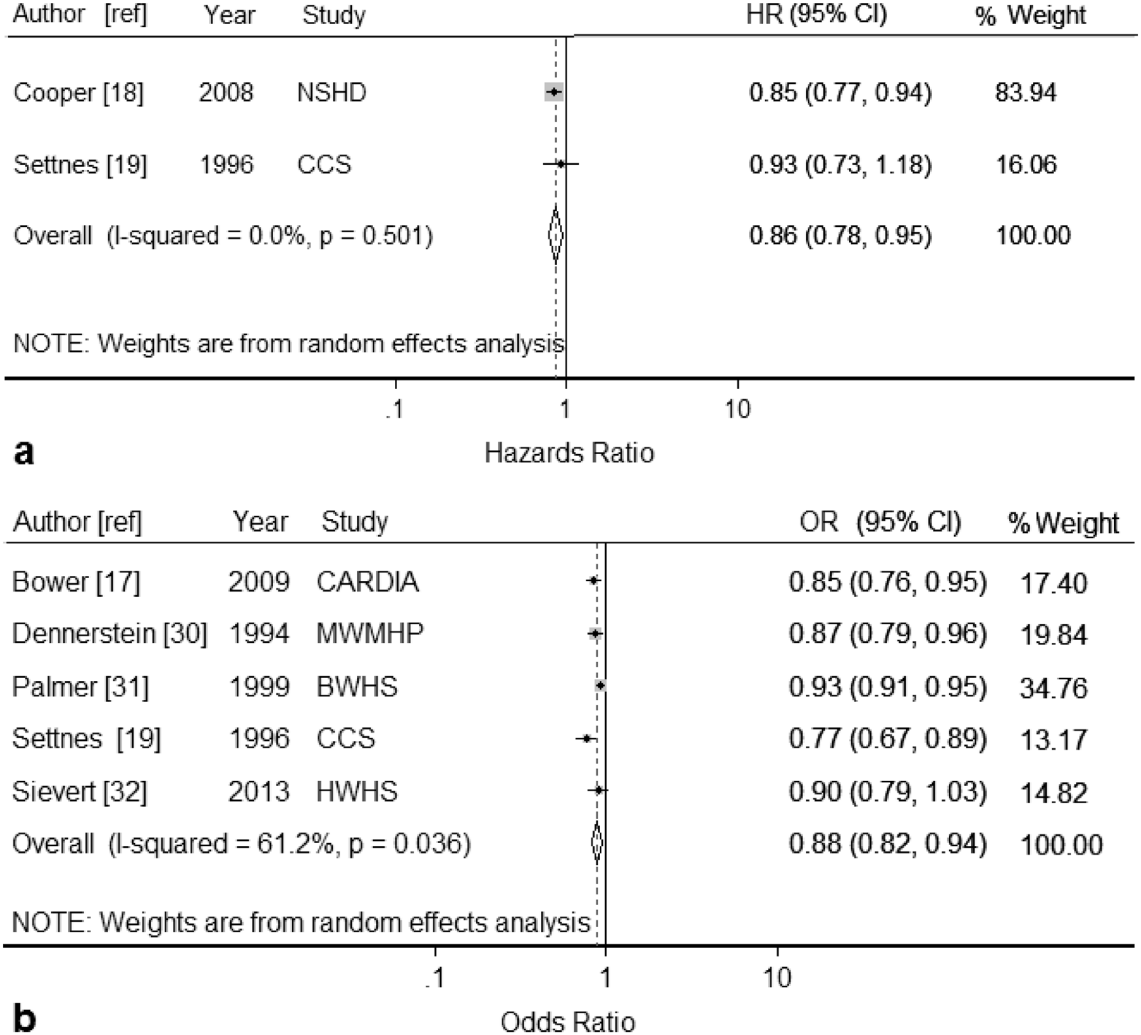
Forest plots displaying results from meta-analyses of the association between each year older at menarche and hysterectomy (random-effects model) for studies reporting: (a) hazard ratios (HR) and, (b) odds ratios (OR). Squares represent study-specific estimates (the size of the square reflects the study-specific statistical weight); horizontal lines represent 95% confidence intervals (CI); diamonds represent the summary estimate with corresponding 95% confidence interval. See Table 1 for details of Study abbreviations.

Seven study populations [17–19, 30–32] were eligible for inclusion in the dose-response meta-analysis. Three study populations [17, 30, 32] reported a dose-response estimate (per year older at menarche) and four [18, 19, 31] reported categorical variables (with the number of categories either 3 or 4). The summary result for the two studies reporting hazard ratios [18, 19] was 0.86 (95% CI: 0.78, 0.95; *I*^*2*^ = 0.0%) per year older at menarche (Fig 2). Random effects meta-analysis of the five cross-sectional studies reporting odds ratios [17, 19, 30–32] gave a summary estimate of 0.88 (95% CI: 0.82, 0.94; *I*^*2*^ = 61%) per year older at menarche (Fig 2). Omitting the Danish Study[19] reduced heterogeneity to 27%, while omitting the results from the Black Women’s Health Study[31] removed all heterogeneity; however in both cases there was minimal difference to the summary result.

Publication bias was not performed as there were insufficient included studies to properly assess a funnel plot or the results of formal assessments.

### Level of Education

#### Lowest versus Highest education level meta-analysis

**Fig 3:**
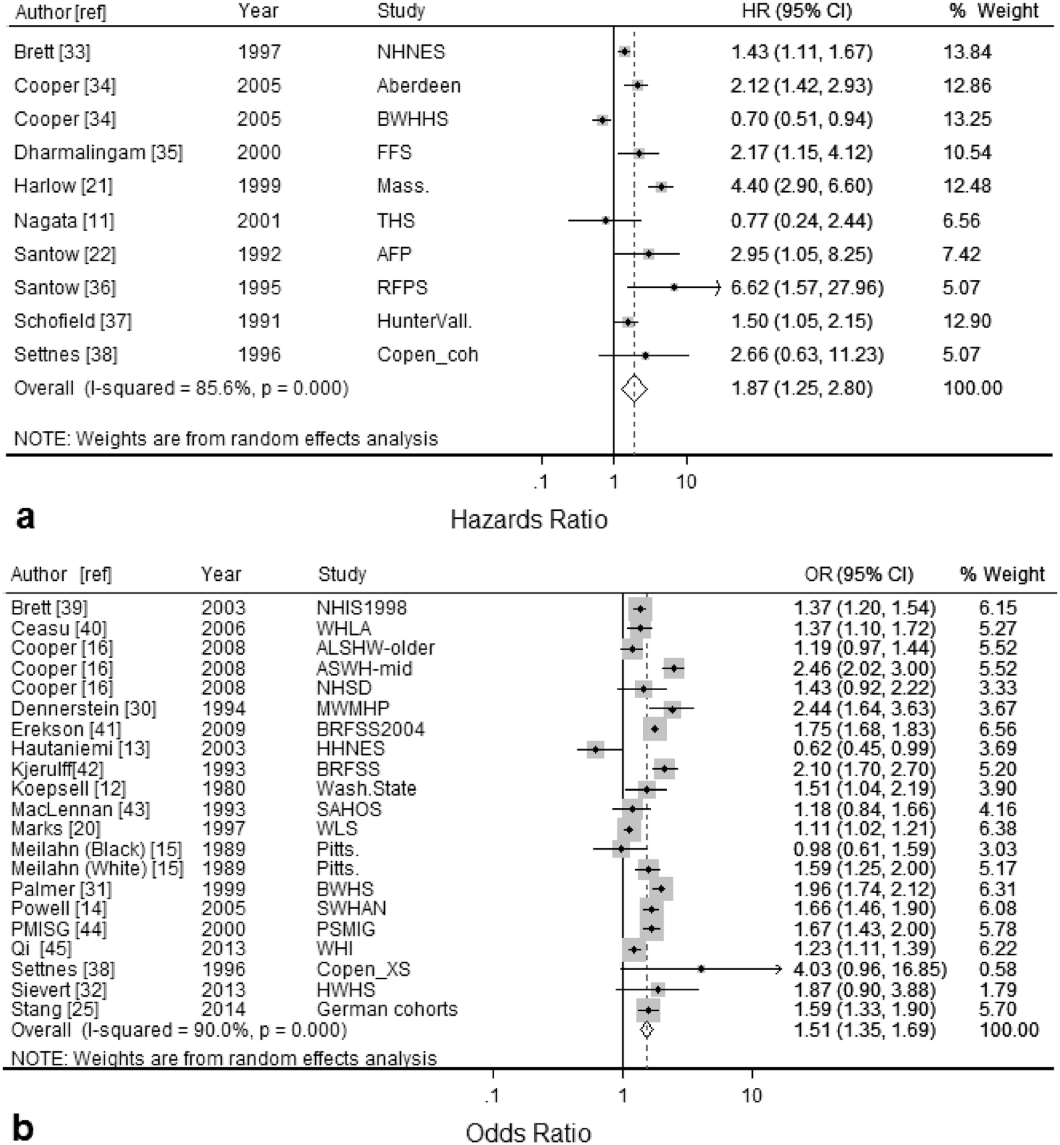
Forest plots displaying results from meta-analyses of the association between level of education and hysterectomy (random-effects model) comparing lowest versus highest education level for studies reporting: (a) hazard ratios (HR) and, (b) odds ratios (OR). Squares represent study-specific estimates (the size of the square reflects the study-specific statistical weight); horizontal lines represent 95% confidence intervals (CI); diamonds represent the summary estimate with corresponding 95% confidence interval. See Table 1 for details of Study abbreviations.

Ten studies reported hazard ratios [11, 21, 22, 33–38]. Random-effects meta-analysis gave a summary HR of 1.87 (95% CI: 1.25, 2.80; *I*^*2*^ = 86%) (Fig 3). Results of sub-group analysis and univariable meta-regression are described in Table 2. Together, those characteristics with a p-value of ≤ 0.2 in univariable meta-regression i.e. year of publication, birth cohort category of study participants and the reference category used for education level explained all of the between-study heterogeneity in a multivariable meta-regression model. Begg and Mazumdar’s correlation was 1.07 (continuity corrected), *P*-value = 0.28. Egger’s regression intercept was 0.151 (*P*-value = 0.33). Thus, there was no evidence of publication bias (available online in S4 File: Funnel Plots).

**Table 2:** Exploration of heterogeneity (Random Effects) in lowest versus highest education level and hysterectomy meta-analysis. Abbreviations: CI, Confidence Interval.

	META-ANALYSIS OF STUDIES REPORTING HAZARD RATIOS				META-ANALYSIS OF STUDIES REPORTING ODDS RATIOS			
Sub-group	No. of studies	Summary Hazard Ratio (95% CI)	Residual heterogeneity (I^2^)	P-value^1^	No. of studies	Summary Odds Ratio (95% CI)	Residual heterogeneity (I^2^)	P-value^1^
*Study Design*								
Cohort	3	1.42 (1.16,1.73)	0.0%	0.43	2	1.15 (0.97,1.35)	18.3%	0.32
Cross-sectional	7	2.15 (1.20,3.82)	89.9%		19	1.55 (1.40,1.72)	85.5%	
*Year of publication*								
Before 2000	6	2.45 (1.46,4.01)	82.4%	0.16	9	1.59 (1.23,2.04)	91.7%	0.60
2000 and later	4	1.30 (0.63,2.67)	88.1%		12	1.48 (1.30,1.68)	88.4%	
*Outcome definition*								
Hysterectomy for malignant conditions excluded	1	2.66 (0.63,11.23)	-	0.72	4	1.79 (1.59,2.02)	51.4%	0.21
Malignant conditions included or not specified	9	1.84 (1.21,2.79)	87.1%		17	1.45 (1.26,1.66)	91.0%	
*Prevalence of hysterectomy in study sample*								
< 15%	7	2.20 (1.48,3.30)	68.2%	0.28	4	1.37 (0.82,2.26)	92.0%	0.77
15 to <25%	3	1.36 (0.66, 2.79)	90.0%		9	1.66 (1.49,1.84)	52.4%	
≥ 25%	-	-	-		8	1.39 (1.13,1.72)	95.0%	
*Birth Cohort category of study participants*								
pre-World War II	2	1.01 (0.50,2.03)	93.1%	0.14	8	1.33 (1.16,1.52)	71.3%	0.05
Baby-boomers	3	2.98 (1.65,5.39)	70.7%		5	1.91 (1.62,2.24)	67.8%	
Broad age group	5	1.92 (1.19,3.10)	45.4%		8	1.49 (1.26,1.75)	85.5%	
*Education Reference Category (highest level)*								
Completed college/university degree	5	2.97 (1.96,4.49)	54.8%	0.03	11	1.53 (1.31,1.78)	93.2%	0.46
Finished school	2	1.45 (1.18,1.77)	0.0%		7	1.63 (1.41,1.88)	40.7%	
Other	3	0.97 (0.52,1.80)	80.7%		3	1.15 (0.59,2.26)	94.6%	
*Region of Study*								
United States	2	2.47 (0.82,7.44)	95.7%	0.63	12	1.44 (1.24,1.67)	93.2%	0.67
Europe/United Kingdom	3	1.43 (0.56,3.66)	91.1%		5	1.57 (1.41,1.74)	0.0%	
Asia/Pacific	1	0.77 (0.24,2.45)	-		-	-	-	
Australia/New Zealand	4	2.15 (1.32,3.50)	43.7%		4	1.70 (1.10,2.63)	91.0%	
*Adjustment for key factors*								
Adjusted for age	4	1.21 (0.66, 2.19)	87.3%	0.29	8	1.75 (1.50, 2.05)	85.3%	0.19
Adjusted for age and at least one reproductive factor	5	2.76 (1.45, 5.23)	85.3%		7	1.36 (1.06, 1.73)	90.4%	
Did not adjust for age/reproductive factor	1	2.66 (0.63, 11.23)	-		6	1.36 (1.12, 1.65)	81.8%	
*Quality of study*								
Moderate	7	1.72 (0.98, 3.04)	89.4%	0.52	17	1.58 (1.40, 1.79)	90.8%	0.12
High	3	2.44 (1.03, 5.76)	66.4%		4	1.17 (0.78, 1.74)	87.3%	

^1^ *p-*values obtained from univariable meta-regression models using the Knapp-Hartung method.

Twenty-one studies reported odds ratios [12–16, 20, 25, 30–32, 38–45]. Random-effects meta-analysis gave a summary OR of 1.51 (95% CI: 1.35, 1.69; *I*^*2*^ = 90%) (Fig 3). When studies that only had unadjusted results were excluded from the meta-analysis [12, 14, 20, 43] the summary estimate was similar with little change in heterogeneity (OR = 1.56; 95% CI: 1.39, 1.75; *I*^*2*^ = 87%). Results of sub-group analysis and univariable meta-regression on the full set of studies are described in Table 2. Birth cohort category of study participants, adjustment for key variables and quality of study were significant in the univariable regression analysis. However, when these characteristics were included in multi-variable meta-regression only 28% of between-study variance was explained. Begg and Mazumdar’s correlation was 0.15 (continuity corrected), *P*-value = 0.88. Egger’s regression intercept was -0.85 (*P*-value = 0.44). Thus, there was no evidence of publication bias (available online in S4 File: Funnel Plots).

#### Education Level Dose-response meta-analysis

**Fig 4:**
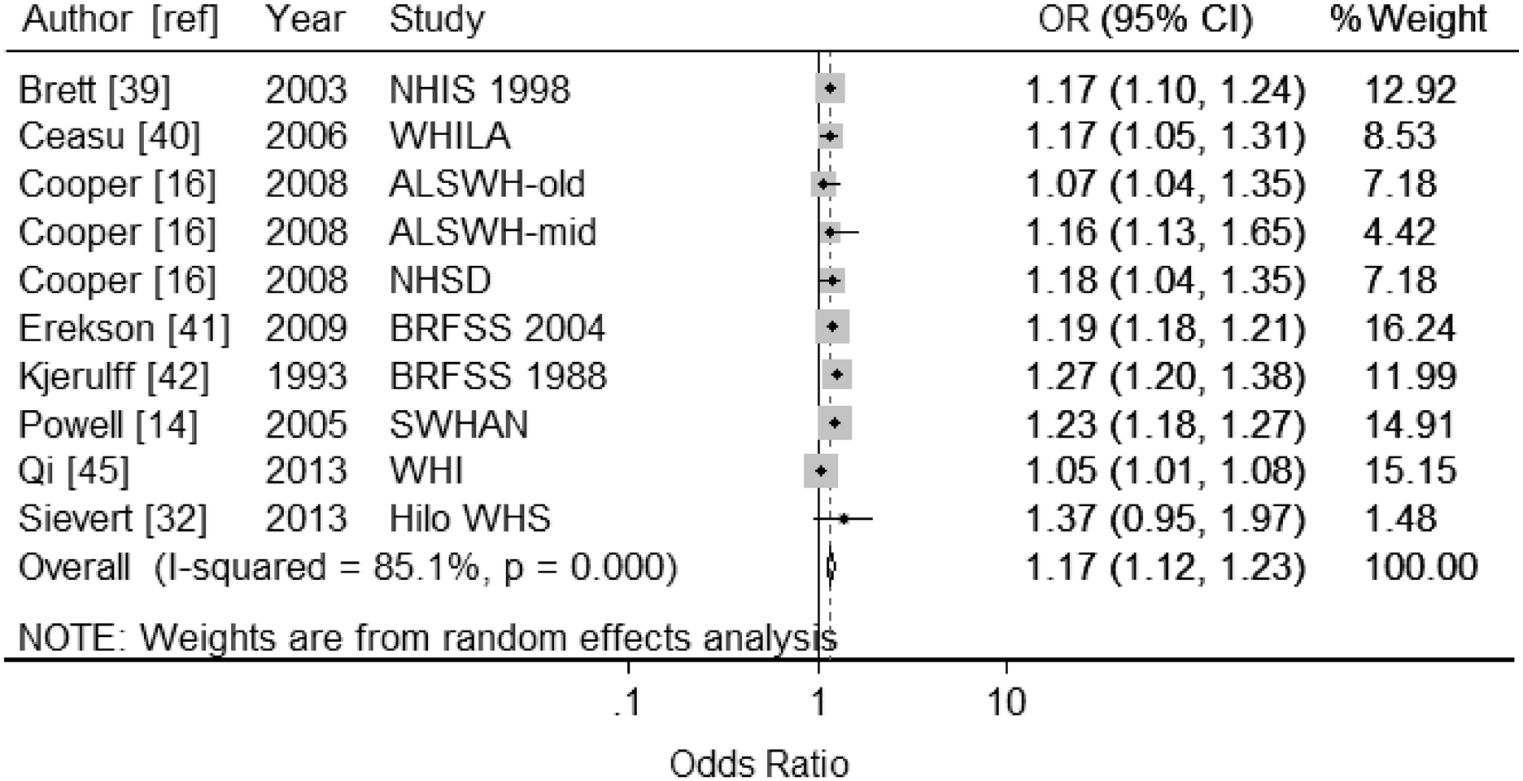
Forest plots displaying results from meta-analyses of the association between each level lower of education and hysterectomy (random-effects model) for studies reporting odds ratios (OR). Squares represent study-specific estimates (the size of the square reflects the study-specific statistical weight); horizontal lines represent 95% confidence intervals (CI); diamonds represent the summary estimate with corresponding 95% confidence interval. See Table 1 for details of Study abbreviations.

Ten studies [14, 16, 32, 39–42, 45] were included in the dose-response meta-analysis. All of the studies reported categorical variables (the average number of categories was 4; ranging from 3 to 6). All of these studies reported odds ratios. Random-effects meta-analysis gave a summary OR of 1.17 (95% CI: 1.12, 1.23; *I*^*2*^ = 85%) per each level lower of education (Fig 4). Results of sub-group analysis are described in Table 3. Together, the birth cohort of study participants and adjustment for at least one reproductive factor explained the heterogeneity between studies. Begg and Mazumdar’s correlation was 0.45 (continuity corrected), *P*-value = 0.65. Egger’s regression intercept was -0.36 (*P*-value = 0.77). Thus, there was no evidence of publication bias (available online in S4 File: Funnel Plots).

**Table 3:** Exploration of heterogeneity (Random Effects) in education level and hysterectomy dose-response meta-analysis. Abbreviations: CI, Confidence Interval.

Sub-group	No. of studies	Summary odds ratio (95% CI)	Residual heterogeneity (I^2^)	P-value^1^
*Study Design*				
Cohort	1	1.18 (1.04, 1.34)	-	.92
Cross-sectional	9	1.17 (1.11, 1.23)	86.7%	
*Year of publication*				
Before 2000	1	1.27 (1.20, 1.38)	-	.21
2000 and later	9	1.16 (1.10, 1.22)	85.7%	
*Outcome definition*				
Hysterectomy for malignant conditions excluded	1	1.23 (1.19, 1.28)	-	.40
Malignant conditions included or not specified	9	1.16 (1.10, 1.23)	85.4%	
*Prevalence of hysterectomy in study sample*				
< 15%	1	1.17 (1.05, 1.31)	-	.27
15 to <25%	5	1.22 (1.19, 1.26)	0.0%	
≥ 25%	4	1.12 (1.01, 1.23)	93.9%	
*Birth Cohort category of study participants*				
pre-World War II	3	1.08 (1.01, 1.15)	41%	.07
Baby-boomers	4	1.23 (1.18, 1.27)	0.0%	
Broad age group	3	1.20 (1.16, 1.24)	44%	
*Region of Study*				
United States	6	1.18 (1.11, 1.25)	91.4%	.45
Europe/United Kingdom	2	1.17 (1.08, 1.28)	0.0%	
Asia/Pacific	-	-	-	
Australia/New Zealand	2	1.10 (0.99, 1.22)	0.0%	
*Adjustment for key factors*				
Adjusted for at least one reproductive factor	8	1.20 (1.17, 1.23)	23.8%	.06
Did not adjust for at least one reproductive factor	2	1.12 (0.90, 1.41)	50.7%	

^1^ *p-*values obtained from univariable meta-regression models using the Knapp-Hartung method. Note: Quality of study not considered in sub-group analysis as all studies were of “moderate” quality.

### Parity

**Fig 5:**
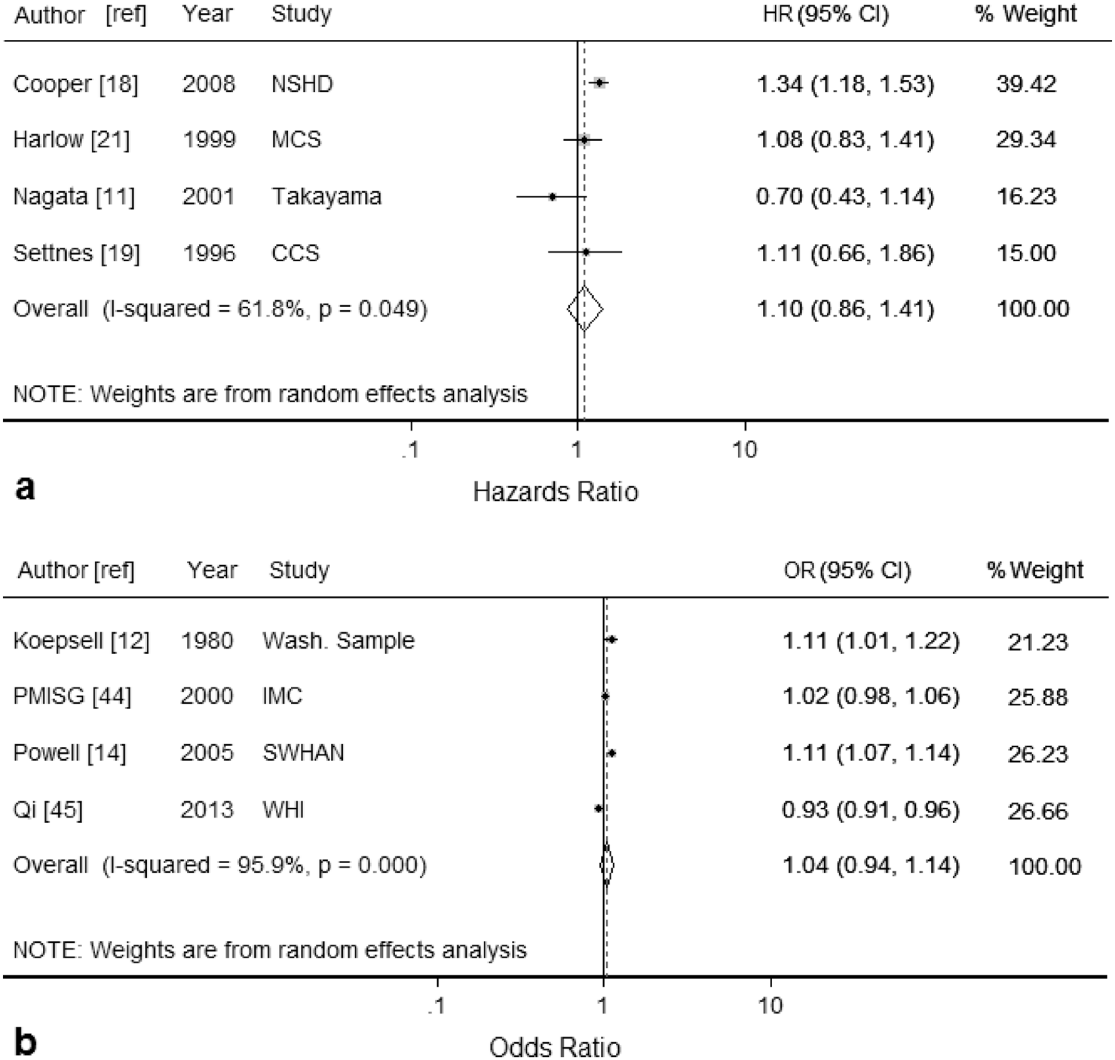
Forest plots displaying results from meta-analyses of the association between each additional child a women gives birth to and hysterectomy (random-effects model) for studies reporting: (a) hazard ratios (HR) and, (b) odds ratios (OR). Squares represent study-specific estimates (the size of the square reflects the study-specific statistical weight); horizontal lines represent 95% confidence intervals (CI); diamonds represent the summary estimate with corresponding 95% confidence interval. See Table 1 for details of Study abbreviations.

Random effects meta-analysis of the four studies [11, 18, 19, 21] reporting hazard ratios gave a non-significant summary estimate of 1.10 (95% CI: 0.86, 1.41; *I*^*2*^ = 62%) per child (Fig 5). Three of the studies, from Japan [11], the USA [21] and Denmark [19], had study populations where the majority of participants were aged less than 45 years (mean age 43, 40 and 35 respectively) when responding to questions about parity. When only these three studies [11, 19, 21] were included in the meta-analysis, heterogeneity was reduced (*I*^2^ = 21%; HR 0.98, 95% CI 0.76, 1.27). When only the Japanese study [11] was omitted (the only study reporting a HR <1), heterogeneity (*I*^*2*^) was 14%, and the association between parity and hysterectomy increased and was statistically significant (HR = 1.26 95% CI 1.09, 1.45 per child). Publication bias was not performed as there were insufficient included studies to properly assess a funnel plot or the results of formal assessments.

Random effects meta-analysis of the four studies [12, 14, 44, 45] reporting odds ratios gave a non-significant summary estimate of 1.04 (95% CI: 0.94, 1.14) per child with high heterogeneity (*I*^*2*^ = 96%) (Fig 5). In the sensitivity analysis, no single study reduced heterogeneity below 80%. Publication bias was not performed as there were insufficient included studies to properly assess a funnel plot or the results of formal assessments.

### Quality analysis

The results of the quality assessments are available online in S5 File: Quality Assessment. Overall, the majority of studies were assessed as being of moderate quality. Main sources of potential bias included: 1) representativeness of the sample in the parity meta-analysis (where a number of studies had young study participants); 2) ascertainment of age at menarche, with only one study[18] obtaining this information during adolescence; 3) comparability of exposed and non-exposed groups, with the majority of studies not adjusting for key confounding variables (parental/early childhood socio-economic factors for age at menarche and reproductive factors for level of education); and 4) assessment of outcome, with the majority of studies ascertaining hysterectomy outcome by self-report and not hospital records or medical imaging.

## Discussion

### Principal Findings

Meta-analyses of the above-mentioned population-based studies revealed a 14% and 12% lower risk of hysterectomy for each year older at menarche in studies reporting hazard ratios and odds ratios respectively. Women with the lowest levels of education had a higher risk of hysterectomy when compared with women with the highest education levels; and there was a dose-response relationship with a 17% higher risk of hysterectomy with each level lower of education. In meta-analyses of studies of parity and hysterectomy, the results (hazard ratios and odds ratios) were not statistically significant.

Early menarche has been associated with an increased risk of uterine fibroids [46–48] and endometriosis [49–51], two common indications for hysterectomy. Although the biologic mechanisms are not clearly understood, it has been postulated that this increased risk may be due to increased hormonal stimulation through increased exposure to menstrual cycles [46, 48, 50], or there may be pre-natal or early life factors that are associated with both early menarche and adult disease [46, 48]. Early menarche may also be a marker for early sexual activity and influence marriage and reproductive patterns, especially age at first birth [52], although the majority of studies adjusted for reproductive variables indicating that an association independent of these factors is present.

Lower levels of education may impact upon general health and health literacy levels, the capacity to navigate the health system and decision-making skills [53] or impact upon the quality of care received through resulting locational or socio-economic disadvantage [54]. Women with lower education levels may work in lower income occupations that may negatively impact on health; or be employed on casual or inflexible terms necessitating quick and complete resolution of medical issues. Additionally, women with lower levels of education may be at increased risk of being overweight and obese (a risk factor for hysterectomy [21, 44, 45, 55] and adjusted for by less than half of the studies included in the education meta-analyses) and having poorer health outcomes overall [56].

### Exploration of heterogeneity

Moderate to high heterogeneity was prevalent in these meta-analyses, in particular, the education level meta-analyses all had high levels of heterogeneity, indicating that the summary results should be interpreted with caution. Sub-group analysis showed that several factors seem to account for this heterogeneity, including the birth cohort category of study participants, the reference category used for level of education, the year the included article was published, quality of the study (as assessed by the authors) and control for the key variables of age and reproductive factors. While most of these explanatory factors relate to the design of the study and measurement of variables, the birth cohort category of study participants as an explanatory factor indicates that education attainment levels may vary over time and may also be a reflection of changing reproductive trends, increased access to alternative treatments for benign hysterectomy indications and changing treatment preferences among different SES groups [16].

Heterogeneity was less of an issue in the age at menarche meta-analyses. There was no heterogeneity in the age at menarche meta-analysis of study populations reporting hazard ratios (although only two studies were able to be included); and in the meta-analysis of study populations reporting odds ratios, the summary result was robust even after omitting studies that showed evidence of contributing to heterogeneity. In the parity meta-analysis of study populations reporting hazard ratios (with moderate heterogeneity) omission of either the UK[18] or Japanese[11] study population reduced heterogeneity to 21% and 14% respectively. In the meta-analysis of study populations reporting odds ratios no single study reduced heterogeneity below 80%; however, in both cases only a small number of studies were able to be included.

### Strengths and limitations

To our knowledge, these are the first meta-analyses to quantify the associations between age at menarche, level of education, parity and risk of hysterectomy. These analyses included only population-based studies to increase generalisability.

Our meta-analyses have several limitations. First, all of the included studies were observational so the results cannot be considered causal evidence. Second, the majority of included studies were cross-sectional with the attendant limitations of this design. Recall bias may have impacted in particular on the accuracy of the measurement of age at menarche. In all but one study [18] this was measured by self-report when women were in adulthood (rather than in adolescence) and less likely to reliably recall menarcheal age [57]. This may have resulted in exposure misclassification although this is unlikely to be differential across hysterectomy and non-hysterectomy groups. Third, in the parity meta-analysis, three of the four studies reporting hazard ratios had populations where the majority of participants were aged less than 45 years at the time of survey, potentially biasing an assessment of the association with hysterectomy (towards the null), as many women delay hysterectomy until they consider their child-bearing years complete.

The process of review and data extraction also demonstrated the differences in outcome and exposure definition across the studies. For example differences in definition and categories in the exposure variables prevented meta-analysis of a number of the SES and adult reproductive factors and contributed to the heterogeneity in the lowest versus highest education meta-analysis. There was very little consistency in adjusting variables across the studies, which also contributed to variance. We anticipated that region of study could have contributed to heterogeneity. This was not the case, however the majority of studies were located in more economically developed countries. Ethnicity may have been a more appropriate measure to assess cultural and contextual differences, however, there was insufficient information across studies to explore this.

### Conclusions and implications

In conclusion, our systematic review and meta-analyses indicate that there is a dose-response relationship between younger age at menarche and lower education levels and a higher risk of hysterectomy. As younger age at menarche, lower education levels and hysterectomy are common risk factors for adverse health outcomes such as diabetes and cardiovascular disease [5, 6, 43, 58–62] a better understanding of the causal pathways and the potential mediating role that hysterectomy might play is of public health importance. Future research needs to explore the relationship between SES and reproductive factors utilising data from large prospective cohort studies that measure SES and reproductive variables prior to hysterectomy and can adjust for key confounding variables. Studies that pool individual-level data from populations of different ethnicities and locations, align outcome and exposure definitions, and can adjust for the same set of confounders are also required.

## Supporting Information

S1 File Search Strategy. This file provides full details of the three search strategies undertaken in Pubmed and Embase.
(PDF)

S2 File Quality Assessment Scales. This file includes the Newcastle-Ottawa Quality Assessment Scales used for each meta-analysis.
(PDF)

S3 File Tables. This file includes details of the studies that reported data on relevant associations but were not included in the meta-analyses.
(PDF)

S4 File Funnel Plots. This file includes the funnel plots assessing publication bias.
(PDF)

S5 File Quality Assessment. This file includes the results of the quality assessments undertaken for each meta-analysis
(PDF)

S6 File PRISMA Checklist. (PDF)
