# Supplementary material for: Age at Menarche, Level of Education, Parity and the Risk of Hysterectomy: A Systematic Review and Meta-Analyses of Population-Based Observational Studies
Source: PLoS One. 2016 Mar 10;11(3):e0151398. doi: 10.1371/journal.pone.0151398 (PMC4786144; doi:10.1371/journal.pone.0151398)
Supplement: S1 File — This file provides full details of the three search strategies undertaken in Pubmed and Embase. (PDF) [file pone.0151398.s001.pdf]

Search strategy.

Search Terms

Searches were conducted in Embase and PubMed for records from inception until March 2015. For all searches we used both Medical Subject Headings (MeSH terms) and text words in PubMed, and Emtree terms and text words in Embase. PubMed Search terms were as follows:

Age at menarche:

(("puberty"[MeSH Terms] OR "puberty"[All Fields]) OR ("menstruation"[MeSH Terms] OR "menstruation"[All Fields]) OR ("menarche"[MeSH Terms] OR "menarche"[All Fields])) AND (("hysterectomy"[MeSH Terms] OR "hysterectomy"[All Fields]) OR ("ovariectomy"[MeSH Terms] OR "ovariectomy"[All Fields] OR "oophorectomy"[All Fields]) OR ("ovariectomy"[MeSH Terms] OR "ovariectomy"[All Fields]))

Socio-economic factors and hysterectomy:

(("education"[Subheading] OR "education"[All Fields] OR "educational status"[MeSH Terms] OR ("educational"[All Fields] AND "status"[All Fields]) OR "educational status"[All Fields] OR "education"[All Fields] OR "education"[MeSH Terms]) OR ("occupations"[MeSH Terms] OR "occupations"[All Fields] OR "occupation"[All Fields]) OR ("income"[MeSH Terms] OR "income"[All Fields]) OR ("poverty"[MeSH Terms] OR "poverty"[All Fields]) OR ("socioeconomic factors"[MeSH Terms] OR ("socioeconomic"[All Fields] AND "factors"[All Fields]) OR "socioeconomic factors"[All Fields]) OR ("employment"[MeSH Terms] OR "employment"[All Fields]) OR ("housing"[MeSH Terms] OR "housing"[All Fields]) OR ("social conditions"[MeSH Terms] OR ("social"[All Fields] AND "conditions"[All Fields]) OR "social conditions"[All Fields])) AND (("hysterectomy"[MeSH Terms] OR "hysterectomy"[All Fields]) OR ("ovariectomy"[MeSH Terms] OR "ovariectomy"[All Fields] OR "oophorectomy"[All Fields]) OR ("ovariectomy"[MeSH Terms] OR "ovariectomy"[All Fields]))

Adult reproductive factors:

(("parity"[MeSH Terms] OR "parity"[All Fields]) OR ("pregnancy"[MeSH Terms] OR "pregnancy"[All Fields]) OR "age at first birth"[All Fields] OR ("gravidity"[MeSH Terms] OR "gravidity"[All Fields]) OR ("abortion, spontaneous"[MeSH Terms] OR ("abortion"[All Fields] AND "spontaneous"[All Fields]) OR "spontaneous abortion"[All Fields] OR ("abortion"[All Fields] AND "spontaneous"[All Fields]) OR "abortion, spontaneous"[All Fields]) OR ("abortion, spontaneous"[MeSH Terms] OR ("abortion"[All Fields] AND "spontaneous"[All Fields]) OR "spontaneous abortion"[All Fields] OR "miscarriage"[All Fields]) OR cesarean[All Fields]) AND (("hysterectomy"[MeSH Terms] OR "hysterectomy"[All Fields]) OR ("ovariectomy"[MeSH Terms] OR "ovariectomy"[All Fields] OR "oophorectomy"[All Fields]) OR ("ovariectomy"[MeSH Terms] OR "ovariectomy"[All Fields])) AND "humans"[MeSH Terms]
