# Supplementary material for: Age at Menarche, Level of Education, Parity and the Risk of Hysterectomy: A Systematic Review and Meta-Analyses of Population-Based Observational Studies
Source: PLoS One. 2016 Mar 10;11(3):e0151398. doi: 10.1371/journal.pone.0151398 (PMC4786144; doi:10.1371/journal.pone.0151398)
Supplement: S2 File — This file includes the Newcastle-Ottawa Quality Assessment Scales used for each meta-analysis. (PDF) [file pone.0151398.s002.pdf]

Figure 1 NEWCASTLE - OTTAWA Quality Assessment Scale

Age at menarche and hysterectomy systematic review and meta-analysis

Note: A study can be awarded a maximum of one star for each numbered item within the Selection and Outcome categories. A maximum of two stars can be given for Comparability. A maximum of nine (9) stars can be awarded to cohort studies; six (6) stars to cross-sectional studies.

Selection

1) Representativeness of the exposed cohort
    - A) Truly representative of average **postmenstrual** women in the community ❋
    - B) Somewhat representative of average **postmenstrual** women in the community ❋
    - C) Selected group of users e.g. nurses, volunteers
    - D) No description of the derivation of the cohort

2) Selection of the non-exposed cohort
    - A) Drawn from the same community as the exposed cohort ❋
    - B) Drawn from a different source
    - C) No description of the derivation of the non-exposed cohort

3) Ascertainment of exposure
    - A) Age at menarche reported during adolescence and confirmed by medical practitioner/nurse or parent ❋
    - B) Age at menarche reported during adolescence ❋
    - C) Written self-report of age at menarche when respondent is an adult
    - D) No description of when report of age at menarche took place

4) Demonstration that outcome of interest was not present at start of study (NOT APPLICABLE TO CROSS-SECTIONAL STUDIES)
    - A) Yes ❋
    - B) No

Comparability

1) Comparability of cohorts on the basis of the design or analysis
    - A) Study controls for at least one **parental/early childhood socio-economic factor** (e.g. father's occupational status). ❋
    - B) Study controls also controls for at least one **reproductive factor** (e.g. age at first birth, parity). ❋

Outcome

1) Assessment of outcome
    - A) Medical imaging ❋
    - B) Record linkage or medical practitioner confirmation ❋
    - C) Self report
    - D) No description
2) Was follow-up long enough for outcomes to occur (NOT APPLICABLE TO CROSS-SECTIONAL STUDIES)
    - A) Yes (as highest incidence rates of hysterectomy occur in women aged 40-49 years, participants should be followed-up to at least age 50) ❋
    - B) No
3) Adequacy of follow up of cohorts (NOT APPLICABLE TO CROSS-SECTIONAL STUDIES)
    - A) Complete follow up - all subjects accounted for ❋
    - B) Subjects lost to follow up unlikely to introduce bias - small number lost (< 20 %) to follow up, or description provided of those lost) ❋
    - C) Loss to follow up rate of >20% and no description of those lost
    - D) No statement

Figure 2 NEWCASTLE - OTTAWA Quality Assessment Scale

Level of education and hysterectomy systematic review and meta-analysis

Note: A study can be awarded a maximum of one star for each numbered item within the Selection and Outcome categories. A maximum of two stars can be given for Comparability. A maximum of nine (9) stars can be awarded to cohort studies; six (6) stars to cross-sectional studies.

Selection

1) Representativeness of the exposed cohort
    - A) Truly representative of average women **who have completed their education** in the community ❋
    - B) Somewhat representative of average women **who have completed their education** in the community ❋
    - C) Selected group of users e.g. nurses, volunteers
    - D) No description of the derivation of the cohort

2) Selection of the non-exposed cohort
    - A) Drawn from the same community as the exposed cohort ❋
    - B) Drawn from a different source
    - C) No description of the derivation of the non-exposed cohort

3) Ascertainment of exposure
    - A) Through structured interview ❋
    - B) Written self-report
    - C) No description

4) Demonstration that outcome of interest was not present at start of study (NOT APPLICABLE TO CROSS-SECTIONAL STUDIES)
    - A) Yes ❋
    - B) No

Comparability

1) Comparability of cohorts on the basis of the design or analysis
    - A) Study controls for **age at survey**. ❋
    - B) Study controls also controls for at least one **reproductive factor** (e.g. age at first birth, parity). ❋

Outcome

1) Assessment of outcome
    - A) Medical imaging ❋
    - B) Record linkage or medical practitioner confirmation ❋
    - C) Self report
    - D) No description
2) Was follow-up long enough for outcomes to occur (NOT APPLICABLE TO CROSS-SECTIONAL STUDIES)
    - A) Yes (as highest incidence rates of hysterectomy occur in women aged 40-49 years, participants should be followed-up to at least age 50) ❋
    - B) No
3) Adequacy of follow up of cohorts (NOT APPLICABLE TO CROSS-SECTIONAL STUDIES)
    - A) Complete follow up - all subjects accounted for ❋
    - B) Subjects lost to follow up unlikely to introduce bias - small number lost (< 20 %) to follow up, or description provided of those lost) ❋
    - C) Loss to follow up rate of >20% and no description of those lost
    - D) No statement

Figure 3 NEWCASTLE - OTTAWA Quality Assessment Scale

Parity and hysterectomy systematic review and meta-analysis

Note: A study can be awarded a maximum of one star for each numbered item within the Selection and Outcome categories. A maximum of two stars can be given for Comparability. A maximum of nine (9) stars can be awarded to cohort studies; six (6) stars to cross-sectional studies.

Selection

1) Representativeness of the exposed cohort
   - A) Truly representative of average women who have completed child-bearing in the community ❋
   - B) Somewhat representative of average women **who have completed child-bearing** in the community ❋
   - C) Selected group of users e.g. nurses, volunteers
   - D) No description of the derivation of the cohort
   - E) Not representative

2) Selection of the non-exposed cohort
   - A) Drawn from the same community as the exposed cohort ❋
   - B) Drawn from a different source
   - C) No description of the derivation of the non-exposed cohort
   - D) Not representative

3) Ascertainment of exposure
   - A) Through structured interview ❋
   - B) Written self-report
   - C) No description

4) Demonstration that outcome of interest was not present at start of study (NOT APPLICABLE TO CROSS-SECTIONAL STUDIES)
   - A) Yes ❋
   - B) No

Comparability

1) Comparability of cohorts on the basis of the design or analysis
   - A) Study controls for **age at survey**❋
   - B) Study controls also controls for **education** ❋

Outcome

1) Assessment of outcome
   - A) Medical imaging ❋
   - B) Record linkage or medical practitioner confirmation ❋
   - C) Self report
   - D) No description
2) Was follow-up long enough for outcomes to occur (NOT APPLICABLE TO CROSS-SECTIONAL STUDIES)
   - A) Yes (as highest incidence rates of hysterectomy occur in women aged 40-49 years, participants should be followed-up to at least age 50) ❋
   - B) No
3) Adequacy of follow up of cohorts (NOT APPLICABLE TO CROSS-SECTIONAL STUDIES)
   - A) Complete follow up - all subjects accounted for ❋
   - B) Subjects lost to follow up unlikely to introduce bias - small number lost (< 20 %) to follow up, or description provided of those lost) ❋
   - C) Loss to follow up rate of >20% and no description of those lost
   - D) No statement
