# Supplementary material for: Age at Menarche, Level of Education, Parity and the Risk of Hysterectomy: A Systematic Review and Meta-Analyses of Population-Based Observational Studies
Source: PLoS One. 2016 Mar 10;11(3):e0151398. doi: 10.1371/journal.pone.0151398 (PMC4786144; doi:10.1371/journal.pone.0151398)
Supplement: S3 File — This file includes details of the studies that reported data on relevant associations but were not included in the meta-analyses. (PDF) [file pone.0151398.s003.pdf]

S3 Tables

Table 1 Summary of Studies Reporting Associations Between Age at Menarche and Hysterectomy, but not Included in Age at Menarche Meta-analysis

Author	Design	Country/data source	Baseline/ Survey year	Age Group (birth cohort)	Sample (% hysterectomy)	Outcome/ comparator	Age at menarche HR/OR (95% CI)	Adjusting variables
Harlow (1999)[1]	cross-sectional	United Stated<br>Random community survey<br>Massachusetts	1995-1997	36-44 yrs<br>(1951-1959)	4,278 (2.66%)	women who were surgically menopausal/women who were premenopausal	Category HR<br>8-10 yrs 1.7 (0.9 - 3.3)<br>11-14 yrs 1.0 (ref)<br>15-17 yrs 0.8 (0.4 - 1.6)	age and other factors (not specified, but other factors in table were age, race, marital status, bmi, smoking, age at menarche, history of irregular cycles, parity, oc use, history of pain with periods, history of endometriosis, history of uterine fibroids, removal of one ovary, combination of endometriosis, uterine fibroids or ovary removal)
Meilahn (1989)[2]	cross-sectional	United States<br>Random telephone survey<br>Pittsburgh	1983	40-52 yrs<br>(1931-1943)	Black participants<br>326 (46.90%)<br>White participants<br>1,785 (23.70%)	Women with a hysterectomy with or without oophorectomy, or oophorectomy along/women without hysterectomy or oophorectomy	Black participants<br>Category OR<br>< 12 yrs 1.0 (ref)<br>≥ 12 yrs 0.68(0.52 - 0.89)<br><br>White participants<br>Category OR<br>< 12 yrs 1.0 (ref)<br>≥ 12 yrs 0.63 (0.37 - 1.07)	age, race, age at menarche, no. of children, BMI, cigarette smoking, religion

Abbreviations: HR, hazard ratio; OR, odds ratio; CI, confidence interval

Table 2 Summary of Studies Reporting Associations Between Level of Education and Hysterectomy, but not Included in Level of Education Meta-analyses

Author	Design	Country/data source	Baseline/ Survey year	Age Group (birth cohort)	Sample (% hysterectomy)	Outcome/ comparator	Education HR/OR (95% CI)	Adjusting variables
Bower (2008)[3]	Cross-sectional	United States<br>Coronary Artery Risk Development in Young Adults (CARDIA) study	1985-1986	18-30 yrs<br>(1955-1967)	1835(10.8%)	Women with a hysterectomy with or without oophorectomy/ Women without a hysterectomy	OR 0.93 (0.86-1.00) per year of education	race, age, geographic site, age at menarche, access to medical care, ability to pay for medical care, depressive symptoms, BMI, polycystic ovarian syndrome, tubal ligation

Abbreviations: HR, hazard ratio; OR, odds ratio; CI, confidence interval

Table 3 Summary of Studies Reporting Associations Between Parity and Hysterectomy, but not Included in Parity Meta-analysis

Author	Design	Country/data source	Baseline/ Survey year	Age range (birth cohort)	Sample (% hysterectomy)	Outcome/ comparator	Parity RR/HR/OR (95% CI)	Adjusting variables
Brett (1997)[4]	cohort	United States<br>National Health and Nutrition Survey	1971-1975 (7-11 yrs follow-up)	<50 yrs (1922-1946)	3526 (22%)	Women with a hysterectomy/Women without a hysterectomy	Also association between pregnancy (no. of live births) and women with hysterectomy for prolapse<br>0-1 RR 0.4 (0.2-0.8)<br>Not significant for other indications	Age, race, education, age at first birth, no. of miscarriages
Dharmalingam (2000)[5]	cross-sectional	Australia<br>Family Formation Survey	1995	25-59 yrs (1936-1975)	2,367 (10.65%)	Women with a hysterectomy / women without a hysterectomy [oophorectomy not mentioned]	No. of children RRs<br>0-1 1.00<br>2-3 1.78 (1.14-2.78)<br>≥ 4 2.04 (1.28-3.24)	calendar period of hysterectomy, age, ethnicity, parity, pregnancy loss, tubal sterilization, use of pill, use of IUD, IUD side effects, educational attainment, occupation, marital status
Hautaniemi (2003)[6]	cross-sectional	United States<br>Hispanic Health and Nutrition Examination Survey	1982-1984	20-74 yrs (1908-1962)	1868 (14.00%)	women with a history of hysterectomy (including with oophorectomy)/ women without a hysterectomy	Parity (ever pregnant)<br>never pregnant 1.00<br>ever pregnant 3.972 (p <0.05)	Base model: adjusted for age, language preference, education, poverty
Marks (1997)[7]	cohort	United States<br>Wisconsin Long Study	1957 (36 yrs follow-up)	53-54 yrs (1939-1940)	3326 (32.1%)	Women who had undergone a hysterectomy by age 54/women without a hysterectomy by age 54	Adjusted ORs<br>0 0 .86 (p ≤ .10)<br>1 1.00<br>2 0.98<br>3 0.92<br>4 0.82 (p ≤ 0.05)<br>5 0.82 (p ≤ 0.05)<br>(confidence intervals unable to be calculated)	father's education, mother's education, father's occupation status 1957, parent's income 1957, respondent's education, mental ability, respondent's occupation status 1975, spouse's occupation status 1975, respondent's income 1974, spouse's income 1974, net worth 1993, married 1993, own home 1993, no. of children, age at first birth
Meilahn (1989)[2]	cross-sectional	United States<br>Random telephone survey<br>Pittsburgh	1983	40-52 yrs (1931-1943)	Black participants 326 (46.90%)<br>White participants 1,785 (23.70%)	Women with a hysterectomy with or without oophorectomy, or oophorectomy alone/ women without hysterectomy or oophorectomy	Black participants:<br>No. of children OR<br>None 1.00<br>One or more 0.22 (0.09-0.55)<br><br>White participants:<br>None 1.00<br>One or more 1.14 (0.81 – 1.59)	age, race, age at menarche, BMI, cigarette smoking, religion
Ong (2000)[8]	Cross-sectional	Ireland	1989	50-65 yrs (1924-1939)	17,735 (22.2%)	Women with a hysterectomy (oophorectomy status unspecified)/ women without a hysterectomy	No. of pregnancies OR<br>None 1.00<br>1-3 1.28 (1.14-1.44)<br>4-6 1.53 (1.37-1.71)<br>7+ 1.61 (1.43-1.82)	Unadjusted ORs only (from raw counts)
Palmer (1999)[9]	cross-sectional	United States<br>Black Women's Health Study	1995	30-49 yrs (1946-1965)	34,950 (14.77%)	Women with a hysterectomy (including oophorectomy) /pre-menopausal women without a hysterectomy. Women with cancer of the cervix or uterus were excluded.	Parity OR<br>0 1.30 (1.10 – 1.54)<br>1 1.33 (1.14 - 1.55)<br>2 1.34 (1.16 – 1.55)<br>3 1.21 (1.03 - 1.41)<br>≥ 4 1.00	current age, geographic region, education, uterine leiomyoma, endometriosis, age at menarche, age at first birth, parity, tubal ligation
Santow (1992)[10]	cross-sectional	Australia<br>Australian Family Project	1986	20-59 yrs (1927-1966)	2,547 (9.70%)	Women with prior history of hysterectomy/ women with intact uteri	Parity<br>Adjusted HRs<br>0-3 1.00<br>3-4 1.71 (1.29 – 2.27)<br>5+ 2.60 (1.70 – 3.97)	age group, parity, side effects IUD, use of pill, tubal sterilization, education, race, state, time period

S3 Tables

Author	Design	Country/data source	Baseline/ Survey year	Age range (birth cohort)	Sample (% hysterectomy)	Outcome/ comparator	Parity RR/HR/OR (95% CI)	Adjusting variables
Santow (1995)[11]	cross-sectional	Australia<br>3rd Risk Factor Prevalence Survey (Canberra component)	1989	20-69 yrs<br>(1929-1969(	276 (16.00%)	Women with a hysterectomy/ women without a hysterectomy	Parity<br>Adjusted HRs<br>0-2 1.00<br>3-4 1.31 (0.64 – 2.67)<br>5-8 2.65 (1.06 – 6.63)	age group, parity, side effects IUD, tubal sterilization, education, caesareans, menstrual problems [model C3]
Settnes (1997)[12]	cross-sectional	Denmark<br>Copenhagen County Random Survey	1982-1984	30, 40, 50, 60 yrs<br>(1922, 1932, 1942, 1952)	1,765 (8.90%)	women with a hysterectomy for benign conditions/ women without a hysterectomy (women with hysterectomy with malignant diagnosis were excluded)	Age-Adjusted ORs<br>1-3 1.00<br>0 1.40 (0.70 - 2.80)<br>>4 2.56 (1.34 – 4.89)<br><br>Adjusted ORs<br>1-3 1.00<br>0 1.10 (0.52 - 2.35)<br>>4 1.49 (0.74 – 3.01)	age, years of general education, vocational education, ascendant social status by marriage
Sievert (2013)[13]	cross-sectional	United States<br>Hilo Women's Health Study	2005	40-60 yrs<br>(1945-1965)	898 (18.37%)	Women with a history of hysterectomy / women without a hysterectomy	Adjusted ORs<br>N/parous 1.00<br>Parous 1.377 (0.682 – 2.780)	age, ethnicity, age at menarche, BMI at age 30, married 20 years ago, parity, education, current smoking

Abbreviations: RR, relative risk; HR, hazard ratio; OR, odds ratio; CI, confidence interval

References

1. Harlow BL, Barbieri RL (1999) Influence of education on risk of hysterectomy before age 45 years. *Am J Epidemiol* 150: 843-847.

2. Meilahn EN, Matthews KA, Egeland G, Kelsey SF (1989) Characteristics of women with hysterectomy. *Maturitas* 11: 319-329.

3. Bower JK, Schreiner PJ, Sternfeld B, Lewis CE (2009) Black-White differences in hysterectomy prevalence: the CARDIA study. *Am J Public Health* 99: 300-307.

4. Brett KM, Marsh JV, Madans JH (1997) Epidemiology of hysterectomy in the United States: demographic and reproductive factors in a nationally representative sample. *J Womens Health* 6: 309-316.

5. Dharmalingam A, Pool I, Dickson J (2000) Biosocial determinants of hysterectomy in New Zealand. *Am J Public Health* 90: 1455-1458.

6. Hautaniemi SI, Leidy Sievert L (2003) Risk factors for hysterectomy among Mexican-American women in the US southwest. *Am J Hum Biol* 15: 38-47.

7. Marks NF, Shinberg DS (1997) Socioeconomic differences in hysterectomy: the Wisconsin Longitudinal Study. *Am J Public Health* 87: 1507-1514.

8. Ong S, Codd MB, Coughlan M, O'Herlihy C (2000) Prevalence of hysterectomy in Ireland. *International Journal of Gynecology and Obstetrics* 69: 243-247.

9. Palmer JR, Rao RS, Adams-Campbell LL, Rosenberg L (1999) Correlates of hysterectomy among African-American women. *Am J Epidemiol* 150: 1309-1315.

10. Santow G, Bracher M (1992) Correlates of hysterectomy in Australia. *Soc Sci Med* 34: 929-942.

11. Santow G (1995) Education and hysterectomy. *Aust N Z J Obstet Gynaecol* 35: 60-69.

12. Settnes A, Lange AP, Jorgensen T (1997) Gynaecological correlates of hysterectomy in Danish women. *Int J Epidemiol* 26: 364-370.

13. Sievert LL, Murphy L, Morrison LA, Reza AM, Brown DE (2013) Age at menopause and determinants of hysterectomy and menopause in a multi-ethnic community: the Hilo Women's Health Study. *Maturitas* 76: 334-341.
