# Supplementary material for: Age at Menarche, Level of Education, Parity and the Risk of Hysterectomy: A Systematic Review and Meta-Analyses of Population-Based Observational Studies
Source: PLoS One. 2016 Mar 10;11(3):e0151398. doi: 10.1371/journal.pone.0151398 (PMC4786144; doi:10.1371/journal.pone.0151398)
Supplement: S4 File — This file includes the funnel plots assessing publication bias. (PDF) [file pone.0151398.s004.pdf]

S4 Funnel Plots

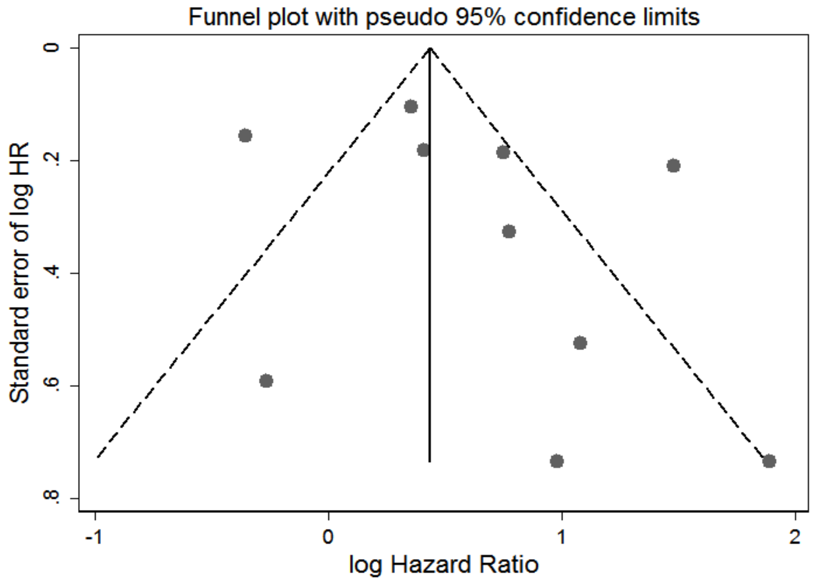

Figure 1 Funnel plot: Lowest versus Highest Education meta-analysis - Studies reporting hazard ratios

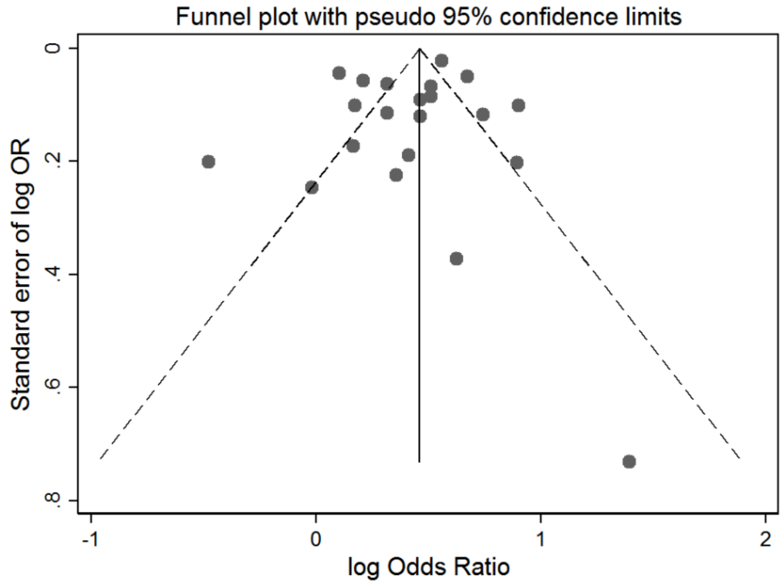

Figure 2 Lowest versus Highest Education meta-analysis - Studies reporting odds ratios

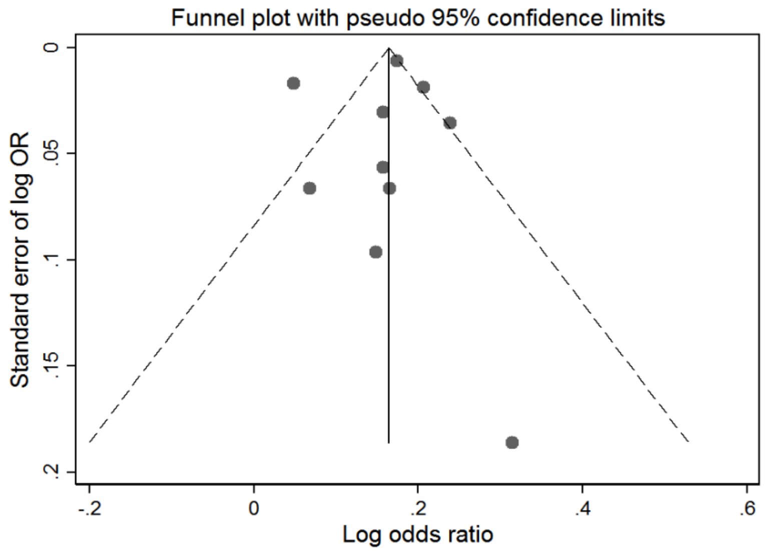

Figure 3 Education dose-response meta-analysis
