# Supplementary material for: Age at Menarche, Level of Education, Parity and the Risk of Hysterectomy: A Systematic Review and Meta-Analyses of Population-Based Observational Studies
Source: PLoS One. 2016 Mar 10;11(3):e0151398. doi: 10.1371/journal.pone.0151398 (PMC4786144; doi:10.1371/journal.pone.0151398)
Supplement: S5 File — This file includes the results of the quality assessments undertaken for each meta-analysis (PDF) [file pone.0151398.s005.pdf]

Table 1 Results of Quality Assessment of Cross-sectional Studies Included in Age at Menarche Meta-analysis

Study	Selection				Comparability		Outcome			Total stars	Quality level
	(1)	(2)	(3)	(4)	(a)	(b)	(1)	(2)	(3)		
Bower (2009)[1] United States Coronary Artery Risk Development in Young Adults (CARDIA) study	B★	A★	C	N/A	No	No	A★	N/A	N/A	3	moderate
Dennerstein (1994)[2] Melbourne Women's Midlife Health Project	B★	A★	C	N/A	No	No	C	N/A	N/A	2	low
Palmer (1999)[3] Black Women's Health Study	B★	A★	C	N/A	No	Yes★	C	N/A	N/A	3	moderate
Settnes (1997)[4] Copenhagen County Study – Cross-sectional	B★	A★	C	N/A	No	Yes★	B★	N/A	N/A	4	moderate
Sievert (2013)[5] Hilo Women's Health Study	B★	A★	C	N/A	No	Yes★	C	N/A	N/A	3	moderate

Table 2 Results of Quality Assessment of Cohort Studies Included in Age at Menarche Meta-analysis

Study	Selection				Comparability		Outcome			Total stars	Quality level
	(1)	(2)	(3)	(4)	(a)	(b)	(1)	(2)	(3)		
Cooper (2008)[6] National Survey of Health & Development	B★	A ★	A ★	A ★	yes ★	yes ★	C	A ★	C	7	high
Settnes (1997)[4] Copenhagen County Study	B★	A★	C	A★	No	Yes★	B★	B	A★	6	moderate

Cross-sectional studies can be awarded a maximum of six stars: We assigned 0-2, 3-4 and 5-6 for low, moderate and high quality studies

Cohort studies can be awarded a maximum of nine stars: we assigned 0-3, 4-6 and 7-9 for low, moderate and high quality studies

Table 3 Results of Quality Assessment of Cross-sectional Studies Included in Level of Education Meta-analyses

Study	In Education meta-analysis		Selection				Comparability		Outcome			Total Stars	Quality level
	Lowest vs. Highest	Dose-response	(1)	(2)	(3)	(4)	(a)	(b)	(1)	(2)	(3)		
Brett (2003)[7] National Health Interview Surveys 1988/1999	Yes	Yes	B★	A★	A★	N/A	Yes★	No	C	N/A	N/A	4	moderate
Ceausu (2006)[8] Women's Health in the Lund Area Survey	Yes	Yes	B★	A★	A★	N/A	No	No	C	N/A	N/A	3	moderate
Cooper (2005)[9] Aberdeen cohort	Yes	Yes	B★	A★	B	N/A	Yes★	No	C	N/A	N/A	3	moderate
Cooper (2005)[9] British Women's Heart and Health Study	Yes		B★	A★	B	N/A	Yes★	No	C	N/A	N/A	3	moderate
Cooper (2008) (8) Australian Longitudinal Study on Women's Health – mid cohort	Yes	Yes	B★	A★	B	N/A	Yes★	No	C	N/A	N/A	3	moderate
Cooper (2008)[10] Australian Longitudinal Study on Women's Health – older cohort	Yes	Yes	B★	A★	B	N/A	Yes★	No	C	N/A	N/A	3	moderate
Dennerstein (1994)[2] Melbourne Women's Midlife Health Project	Yes		B★	A★	A★	N/A	Yes★	No	C	N/A	N/A	4	moderate
Dharmalingham (2000)[11] New Zealand Family Formation Study	Yes		B★	A★	B	N/A	Yes★	Yes★	C	N/A	N/A	4	moderate
Erekson (2009)[12] Behavioral Risk Factor Surveillance Survey 2004	Yes	Yes	B★	A★	A★	N/A	Yes★	No	C	N/A	N/A	4	moderate
Harlow (1999)[13] Postal survey Massachusetts	Yes		B★	A★	B	N/A	Yes★	Yes★	C	N/A	N/A	4	moderate
Hautaniemi (2003)[14] Hispanic Health and Nutrition Examination Survey	Yes		B★	A★	A★	N/A	Yes★	Yes★	C	N/A	N/A	5	high
Kjerulff (1993)[15] Behavioral Risk Factor Surveillance Survey 1988	Yes	Yes	B★	A★	A★	N/A	Yes★	No	C	N/A	N/A	4	moderate
Koepsell (1980)[16] Washington State Sample	Yes		B★	A★	A★	N/A	No	No	C	N/A	N/A	3	moderate
MacLennan (1993)[17] South Australia Health Omnibus Survey	Yes		B★	A★	A★	N/A	No	No	C	N/A	N/A	3	moderate
Meilahn (1989)[18] Random telephone survey Pittsburgh	Yes		B★	A★	A★	N/A	Yes★	Yes★	C	N/A	N/A	5	high
Palmer (1999)[3] Black Women's Health Study	Yes		B★	A★	B	N/A	Yes★	Yes★	C	N/A	N/A	4	moderate
PMISG (2000)[19] Women attending Italian Menopause clinics	Yes		C	A★	A★	N/A	Yes★	Yes★	B★	N/A	N/A	5	high
Powell (2005)[20] Study of Women's Health Across the Nation	Yes	Yes	B★	A★	A★	N/A	No	No	C	N/A	N/A	3	moderate
Qi (2013)[21] Women's Health Initiative	Yes	Yes	B★	A★	B	N/A	Yes★	Yes★	C	N/A	N/A	4	moderate
Santow (1992)[22] Australian Family Project	Yes		B★	A★	A★	N/A	Yes★	Yes★	C	N/A	N/A	5	high
Santow (1995)[23] 3rd Risk Factor Prevalence Survey (Canberra component)	Yes		B★	A★	A★	N/A	Yes★	Yes★	C	N/A	N/A	5	high
Schofield (1991)[24] Hunter Valley Survey	Yes		A★	A★	A★	N/A	Yes★	No	C	N/A	N/A	4	moderate
Settnes (1996)[25] Copenhagen County Study – Cross-sectional	Yes		B★	A★	B	N/A	Yes★	No	B★	N/A	N/A	4	moderate
Sievert (2013)[5] Hilo Women's Health Study	Yes	Yes	B★	A★	B	N/A	Yes★	Yes★	C	N/A	N/A	4	moderate
Stang (2014)[26] Pooled analysis of 6 German cohorts	Yes		B★	A★	B	N/A	Yes★	No	C	N/A	N/A	3	moderate

Table 4 Results of Quality Assessment of Cohort Studies Included in Level of Education Meta-analyses

Study	In Education meta-analysis		Selection				Comparability		Outcome			Total stars	Quality level
	Lowest vs. Highest	Dose-response	(1)	(2)	(3)	(4)	(a)	(b)	(1)	(2)	(3)		
Brett (1997) [27]United States National Health and Nutrition Survey	Yes		B★	A★	A★	A★	Yes★	Yes★	B★	A★	D	8	high
Cooper (2008)[10] National Survey of Health & Development	Yes	Yes	B★	A ★	A★	A ★	No	No	C	A ★	C	5	moderate
Marks (1997)[28] Wisconsin Longitudinal Study	Yes		C	A★	A★	A★	No	No	C	A★	C	4	moderate
Nagata (2001)[29] Takayama Study	Yes		B★	A★	B	A★	Yes★	Yes★	C	B	B★	5	moderate
Settnes (1996)[25] Copenhagen County Study - cohort	Yes		B★	A★	B	A★	No	No	B★	B	A★	5	moderate

Cross-sectional studies can be awarded a maximum of six stars: We assigned 0-2, 3-4 and 5-6 for low, moderate and high quality studies

Cohort studies can be awarded a maximum of nine stars: we assigned 0-3, 4-6 and 7-9 for low, moderate and high quality studies

Table 5 Results of Quality Assessment of Cross-sectional Studies Included in Parity Meta-analysis

Study	Selection				Comparability		Outcome			Total Stars	Quality level
	(1)	(2)	(3)	(4)	(a)	(b)	(1)	(2)	(3)		
Harlow (1999)[13] Postal survey Massachusetts	E	A★	B	N/A	Yes★	Yes★	C	N/A	N/A	3	moderate
Koepsell (1980)[16] Washington State Sample	B★	A★	A★	N/A	No	No	C	N/A	N/A	3	moderate
PMISG (2000)[19] Women attending Italian Menopause clinics	C	A★	A★	N/A	Yes★	Yes★	B★	N/A	N/A	5	high
Powell (2005)[20] Study of Women's Health Across the Nation	B★	A★	A★	N/A	No	No	C	N/A	N/A	3	moderate
Qi (2013)[21] Women's Health Initiative	B★	A★	B	N/A	No	No	C	N/A	N/A	2	low

Table 6 Results of Quality Assessment of Cohort Studies Included in Parity Meta-analysis

Study	Selection				Comparability		Outcome			Total stars	Quality level
	(1)	(2)	(3)	(4)	(a)	(b)	(1)	(2)	(3)		
Cooper (2008)[6] National Survey of Health & Development	B★	A ★	A ★	A ★	No	Yes★	C	A ★	C	6	moderate
Nagata (2001)[29] Takayama Study	E	A★	B	A★	Yes★	Yes★	C	B	B★	5	moderate
Settnes (1997)[4] Copenhagen County Study - cohort	E	A★	B	A★	Yes★	No	B★	B	A★	5	moderate

Cross-sectional studies can be awarded a maximum of six stars: We assigned 0-2, 3-4 and 5-6 for low, moderate and high quality studies
Cohort studies can be awarded a maximum of nine stars: we assigned 0-3, 4-6 and 7-9 for low, moderate and high quality studies

References

1. Bower JK, Schreiner PJ, Sternfeld B, Lewis CE (2009) Black-White differences in hysterectomy prevalence: the CARDIA study. *Am J Public Health* 99: 300-307.

2. Dennerstein L, Shelley J, Smith AM, Ryan M (1994) Hysterectomy experience among mid-aged Australian women. *Med J Aust* 161: 311-313.

3. Palmer JR, Rao RS, Adams-Campbell LL, Rosenberg L (1999) Correlates of hysterectomy among African-American women. *Am J Epidemiol* 150: 1309-1315.

4. Settnes A, Lange AP, Jorgensen T (1997) Gynaecological correlates of hysterectomy in Danish women. *Int J Epidemiol* 26: 364-370.

5. Sievert LL, Murphy L, Morrison LA, Reza AM, Brown DE (2013) Age at menopause and determinants of hysterectomy and menopause in a multi-ethnic community: the Hilo Women's Health Study. *Maturitas* 76: 334-341.

6. Cooper R, Hardy R, Kuh D (2008) Timing of menarche, childbearing and hysterectomy risk. *Maturitas* 61: 317-322.

7. Brett KM, Higgins JA (2003) Hysterectomy prevalence by Hispanic ethnicity: evidence from a national survey. *Am J Public Health* 93: 307-312.

8. Ceausu I, Shakir YA, Lidfeldt J, Samsioe G, Nerbrand C (2006) The hysterectomized woman. Is she special? The women's health in the Lund area (WHILA) study. *Maturitas* 53: 201-209.

9. Cooper R, Lawlor DA, Hardy R, Ebrahim S, Leon DA, et al. (2005) Socio-economic position across the life course and hysterectomy in three British cohorts: a cross-cohort comparative study. *BJOG* 112: 1126-1133.

10. Cooper R, Lucke J, Lawlor DA, Mishra G, Chang JH, et al. (2008) Socioeconomic position and hysterectomy: a cross-cohort comparison of women in Australia and Great Britain. *J Epidemiol Community Health* 62: 1057-1063.

11. Dharmalingam A, Pool I, Dickson J (2000) Biosocial determinants of hysterectomy in New Zealand. *Am J Public Health* 90: 1455-1458.

12. Erekson EA, Weitzen S, Sung VW, Raker CA, Myers DL (2009) Socioeconomic indicators and hysterectomy status in the United States, 2004. *J Reprod Med* 54: 553-558.

13. Harlow BL, Barbieri RL (1999) Influence of education on risk of hysterectomy before age 45 years. *Am J Epidemiol* 150: 843-847.

14. Hautaniemi SI, Leidy Sievert L (2003) Risk factors for hysterectomy among Mexican-American women in the US southwest. *Am J Hum Biol* 15: 38-47.

15. Kjerulff K, Langenberg P, Guzinski G (1993) The socioeconomic correlates of hysterectomies in the United States. *Am J Public Health* 83: 106-108.

16. Koepsell TD, Weiss NS, Thompson DJ, Martin DP (1980) Prevalence of prior hysterectomy in the Seattle-Tacoma area. *Am J Public Health* 70: 40-47.

17. MacLennan AH, MacLennan A, Wilson D (1993) The prevalence of hysterectomy in South Australia. *Med J Aust* 158: 807-809.

18. Meilahn EN, Matthews KA, Egeland G, Kelsey SF (1989) Characteristics of women with hysterectomy. *Maturitas* 11: 319-329.

19. Progetto Menopausa Italia Study Group (PMISG) (2000) Determinants of hysterectomy and oophorectomy in women attending menopause clinics in Italy. *Maturitas* 36: 19-25.

20. Powell LH, Meyer P, Weiss G, Matthews KA, Santoro N, et al. (2005) Ethnic differences in past hysterectomy for benign conditions. *Womens Health Issues* 15: 179-186.

21. Qi L, Nassir R, Kosoy R, Garcia L, Waetjen LE, et al. (2013) Relationship between hysterectomy and admixture in African American women. *Am J Obstet Gynecol* 208: 279 e271-277.

22. Santow G, Bracher M (1992) Correlates of hysterectomy in Australia. *Soc Sci Med* 34: 929-942.

23. Santow G (1995) Education and hysterectomy. *Aust N Z J Obstet Gynaecol* 35: 60-69.

24. Schofield MJ, Hennrikus DJ, Redman S, Sanson-Fisher RW (1991) Prevalence and characteristics of women who have had a hysterectomy in a community survey. *Aust N Z J Obstet Gynaecol* 31: 153-158.

25. Settnes A, Jorgensen T (1996) Hysterectomy in a Danish cohort. Prevalence, incidence and socio-demographic characteristics. *Acta Obstet Gynecol Scand* 75: 274-280.

26. Stang A, Kluttig A, Moebus S, Volzke H, Berger K, et al. (2014) Educational level, prevalence of hysterectomy, and age at amenorrhoea: a cross-sectional analysis of 9536 women from six population-based cohort studies in Germany. *BMC Womens Health* 14: 10.

27. Brett KM, Marsh JV, Madans JH (1997) Epidemiology of hysterectomy in the United States: demographic and reproductive factors in a nationally representative sample. *J Womens Health* 6: 309-316.

28. Marks NF, Shinberg DS (1997) Socioeconomic differences in hysterectomy: the Wisconsin Longitudinal Study. *Am J Public Health* 87: 1507-1514.

29. Nagata C, Takatsuka N, Kawakami N, Shimizu H (2001) Soy product intake and premenopausal hysterectomy in a follow-up study of Japanese women. *Eur J Clin Nutr* 55: 773-777.
